# Cost-effectiveness of Internet Interventions Compared With Treatment as Usual for People With Mental Disorders: Systematic Review and Meta-analysis of Randomized Controlled Trials

**DOI:** 10.2196/38204

**Published:** 2023-01-05

**Authors:** Pieter J Rohrbach, Alexandra E Dingemans, Catharine Evers, Eric F Van Furth, Philip Spinhoven, Jiska J Aardoom, Irene Lähde, Fleur C Clemens, M Elske Van den Akker-Van Marle

**Affiliations:** 1 GGZ Rivierduinen Eating Disorders Ursula Leiden Netherlands; 2 Department of Psychiatry Leiden University Medical Center Leiden Netherlands; 3 Department of Social, Health and Organizational Psychology Utrecht University Utrecht Netherlands; 4 Institute of Psychology Leiden University Leiden Netherlands; 5 Department of Public Health and Primary Care Leiden University Medical Center Leiden Netherlands; 6 Section of Medical Decision Making Department of Biomedical Data Sciences Leiden University Medical Center Leiden Netherlands

**Keywords:** economic evaluation, cost-benefit analysis, methods of economic evaluation, meta-analysis, eHealth, internet-based interventions, mental disorders, mental health, systematic review, randomized controlled trial, mobile phone

## Abstract

**Background:**

The economic costs of mental disorders for society are huge. Internet-based interventions are often coined as cost-effective alternatives to usual care, but the evidence is mixed.

**Objective:**

The aim was to review the literature on the cost-effectiveness of internet interventions for mental disorders compared with usual care and to provide an estimate of the monetary benefits of such interventions compared with usual care.

**Methods:**

A systematic review and meta-analysis of randomized controlled trials was conducted, which included participants with symptoms of mental disorders; investigated a telephone- or internet-based intervention; included a control condition in the form of treatment as usual, psychological placebo, waiting list control, or bibliotherapy; reported outcomes on both quality of life and costs; and included articles published in English. Electronic databases such as PubMed (including MEDLINE), Embase, Emcare, PsycINFO, Web of Science, and the Cochrane Library were used. Data on risk of bias, quality of the economic evaluation, quality-adjusted life years, and costs were extracted from the included studies, and the incremental net benefit was calculated and pooled.

**Results:**

The search yielded 6226 abstracts, and 37 studies with 14,946 participants were included. The quality of economic evaluations of the included studies was rated as moderate, and the risk of bias was high. A random-effects approach was maintained. Analyses suggested internet interventions were slightly more effective than usual care in terms of quality-adjusted life years gain (Hedges *g*=0.052, 95% CI 0.010-0.094; *P*=.02) and equally expensive (Hedges *g*=0.002, 95% CI −0.080 to 0.84; *P*=.96). The pooled incremental net benefit was US $255 (95% CI US $91 to US $419; *P*=.002), favoring internet interventions over usual care. The perspective of the economic evaluation and targeted mental disorder moderated the results.

**Conclusions:**

The findings indicate that the cost-effectiveness of internet interventions for mental disorders compared with a care-as-usual approach is likely, but generalizability to new studies is poor given the substantial heterogeneity. This is the first study in the field of mental health to pool cost-effectiveness outcomes in an aggregate data meta-analysis.

**Trial Registration:**

PROSPERO CRD42019141659; https://tinyurl.com/3cu99b34

## Introduction

### Background

Mental disorders have a big impact on affected individuals as well as on the society. It is appraised to cause almost one-third of global years lived with disability and account for roughly 10% of all disability-adjusted life years, placing it in the top 3 causes of global burden worldwide [1]. When mental, neurological, and substance use disorders were taken together, the global economic costs in 2010 were estimated to be US $2.5 trillion, with projections for 2030 being around US $6 trillion [2]. While these numbers are serious and account for approximately 2.3% to 4.4% of the gross domestic product in high-income countries, most countries spend a disproportionally small amount of their health budget on mental health [3]. This not only warrants changes in policy but also stresses the need for effective and inexpensive interventions so that individuals can recover more swiftly from a mental disorder and the global burden is reduced.

### Effectiveness of Internet Interventions

Swift technological advancement brings the promise of new and effective interventions. Indeed, internet interventions have become a popular niche of research and treatment. A reason for attempting to create effective internet interventions is to reduce the treatment gap, which specifies the discrepancy between the proportion of people who need help for a particular disorder and the proportion of those individuals who actually receive care [4,5]. In other words, internet interventions can be used to reach an underserved population of people with a mental illness or a risk of developing a mental illness [6]. Internet interventions for people with symptoms of a mental disorder are increasingly confirmed in their effectiveness. There have been several meta-analyses on this topic covering various mental disorders such as depression, anxiety, posttraumatic stress, and eating disorders [7-11]. A recent umbrella review of meta-analyses shows that there is sufficient information to assume the effectiveness of internet interventions [12]. In general, guided internet interventions (most of which have a cognitive behavior underpinning) seem to be as effective in reducing symptomatology as face-to-face treatment and outperforming waitlist control conditions. Unguided internet interventions seem to be more effective than waitlist control conditions but less effective than guided internet or face-to-face interventions.

### Cost-effectiveness of Internet Interventions

Internet interventions for mental disorders are often coined as a cost-effective alternative to established treatments [13], but the results on cost-effectiveness are tentative at best [14,15]. Individual studies show mixed results, and their heterogeneity in methods, outcomes, and comparators makes it difficult to draw conclusions [16,17], so no definitive assumptions can be established. In addition, cost-effectiveness studies conducted alongside randomized controlled trials (RCTs) are often not powered for economic evaluations, limiting their predictive ability [18]. Nevertheless, separate studies make an important contribution to the rapidly growing body of evidence, so that it becomes more feasible to make meaningful overviews in the form of systematic reviews and meta-analyses. Indeed, Naslund et al [17] performed a broad systematic review on cost outcomes for telemedicine interventions for mental disorders, and Donker et al [16] systematically reviewed RCTs on the cost-effectiveness of internet-based interventions for mental disorders. Both reviews concluded that internet interventions for mental disorders have the potential to be cost-effective compared with alternatives, but evidence is still circumstantial. Kolovos et al [19] performed an individual participant meta-analysis to investigate the cost-effectiveness of internet interventions compared with a control condition (eg, waiting list or care-as-usual) for depression. The authors cautiously concluded that the results showed no indication of cost-effectiveness and remarked that adding economic evaluations to trials more frequently would help to reach well-founded deductions.

### Pooling Cost-effectiveness Data

To obtain a precise estimate of the cost-effectiveness of internet interventions for mental disorders compared with alternatives, it is desirable to pool the outcomes of individual studies in an aggregate data meta-analysis. As cost-effectiveness is expressed as a combination of 2 variables, the difference in costs and effects between an intervention and control condition, pooling cost-effectiveness outcomes is statistically complex. Fortunately, Crespo et al [20] developed a theoretical framework to perform these kinds of analyses. This method has been successfully applied by another research team in several studies in different areas of medicine [21-25]. Currently, no meta-analysis on the cost-effectiveness of internet interventions for mental disorders has been conducted. The individual participant data meta-analysis by Kolovos et al [19] examined depression only. The individual participant data approach is more reliable [26]; however, it is decreasingly feasible when more studies are included. Furthermore, the last overview of RCTs on the cost-effectiveness of internet-based interventions for mental disorders was conducted in 2015 [16], but the results were not pooled in a meta-analysis.

Concordantly, given the mixed evidence and limitations of individual studies and the rapid increase of novel research, a thorough and recent overview of the literature on the cost-effectiveness of internet interventions for individuals with a mental disorder compared with alternatives is warranted. A first step would be to establish whether internet interventions are cost-effective compared with control conditions such as a waiting list and care-as-usual. In this study, we aimed to investigate the cost-effectiveness of internet interventions for mental disorders compared with control conditions by conducting a systematic literature search and aggregate data meta-analysis of RCTs.

## Methods

The literature review was conducted according to the PRISMA (Preferred Reporting Items for Systematic Reviews and Meta-Analyses) guidelines [27] (Multimedia Appendix 1). This review was registered in PROSPERO (registration number CRD42019141659).

### Eligibility Criteria

The inclusion criteria were studies that (1) were RCTs, (2) included participants (of all ages) who have symptoms of or a diagnosed mental disorder, and (3) investigated a telephone- or internet-based (that work via computer, tablet, or smartphone) intervention. All forms of internet-based interventions were considered, including fully automated (ie, unguided) interventions, guided interventions, and teleconferencing interventions. Guided interventions could contain asynchronous support (ie, a delay in the support such as with mail or forum services) or synchronous support (ie, no delay in the support such as with videoconferencing and chat). Smartphone apps with the purpose of elevating the symptoms of a mental disorder were included. In addition, studies were included in this review that (4) included a control condition in the form of (enhanced) treatment as usual, psychological placebo, waitlist control, or bibliotherapy; (5) included reported outcomes on both quality of life (quality-adjusted life years [QALYs]) and costs; and (6) were published in English. It is worth noting that studies were included if participants had somatic conditions (eg, cancer or diabetes) as long as the participants had comorbid symptoms of a mental disorder, and the investigated intervention had the primary aim to alleviate these symptoms. Studies were excluded if the main intervention relied exclusively on wearable devices or virtual reality, had only a face-to-face intervention as a control condition, or did not provide sufficient information on costs or QALYs.

### Search Strategy and Selection Criteria

The electronic databases PubMed (including MEDLINE), Embase, Emcare, PsycINFO, Web of Science, and the Cochrane Library were searched until March 1, 2021. A search string was created for PubMed and translated for the other databases. The PubMed search string contained Medical Subject Headings terms for the concepts of mental disorders, cost-benefit analysis, telemedicine, and internet, with all expressions under these headings included as free terms as well. Furthermore, other terms related to the 3 Medical Subject Headings terms were added to maximize the sensitivity of the search. The full PubMed search strategy is presented in Item 1 of Multimedia Appendix 2. By checking cross-references in the included studies, we minimized the chance of missing relevant data. During the screening phase, all relevant study protocols, conference abstracts, and trial registrations were identified, and the authors of unpublished studies with potentially relevant data were contacted.

The identified articles were screened in 3 steps by 2 researchers (PJR, AED, CE, MEVA-VM, IL, or FCC). First, titles and abstracts of the eligible articles were screened. Subsequently, full texts of all included abstracts were screened for eligibility. Finally, relevant data were extracted. If there was any disagreement between the 2 researchers in any of the 3 steps, a third researcher from the team made the final decision.

### Quality Assessment

Risk of bias of the included RCTs was assessed by 2 researchers (PJR, AED, CE, IL, or FCC) using the Cochrane Risk of Bias assessment tool [28]. Specifically, data were gathered on the topics of (1) random sequence generation (selection bias), (2) allocation concealment (selection bias), (3) blinding of participants and personnel (performance bias), (4) blinding of outcome assessment (detection bias), (5) incomplete outcome data (attrition bias), (6) selective reporting (reporting bias), and (7) other sources of bias. Finally, for all these topics, the risk of bias was assessed (ie, low, unclear, or high risk of bias). Any disagreement between the researchers on each of the 7 areas of the risk of bias was resolved by means of a discussion between the 2 raters or by a third rater. A final rating of high, medium, or low bias was assigned to each study based on the revised tool for assessing the risk of bias [29]. Specifically, a high risk of bias was assigned to studies when a high risk of bias in any domain was present or unclear risk of bias was present in ≥2 domains, medium risk of bias was assigned in the case of 1 unclear rating across all domains, and low risk of bias was assigned when all domains were rated as low risk of bias. Exceptions were that blinding of participants and personnel was not considered in the final risk of bias rating as blinding was unfeasible in most studies, and unclear risk of bias on the selective reporting domain was considered as low risk of bias for the final risk of bias rating as the absence of a published protocol or trial preregistration is still common.

Furthermore, the 19-item Consensus Health Economic Criteria (CHEC) list [30] was used to assess the quality of the economic evaluation for all included studies. The expert on economic evaluations (MEVA-VM) scored all articles on the CHEC list, while other authors (PJR, AED, CE, IL, and FCC) divided included articles for the CHEC list as well, so articles were rated twice. All discrepancies between raters on the CHEC list were resolved through a discussion. Assigning an overall quality score for the CHEC list is not advocated, as cutoff scores are highly heterogeneous [31] and difficult to substantiate. As we wanted to group studies by the quality of the economic evaluation in analyses, we adopted an approach using a selection of items from the CHEC list. A study had to fulfill at least 8 specific items (ie, items 7-14, see *Risk of Bias and Quality of Economic Evaluation* below for a list of all items) to be deemed of high quality. These items were chosen because they contribute to the assessment of the costs and effects used in the current meta-analysis. Other items (ie, 1-4 and 15-19) were deemed less important for this study, as they aim at clarifying the text and the appropriateness of the used methods and analyses, which is also captured partly in the risk of bias assessment. Finally, items 5 and 6 regarding time horizon and perspective were not considered for the final quality rating because they were already explored in the moderator analyses.

### Outcome Measures

#### Quality of Life

The difference in the average number of QALYs gained per participant in the intervention group and its comparator (delta QALY) was the target health outcome measure. A QALY indicates 1 extra life-year in perfect health. QALYs are derived from generic health-related quality-of-life measures (eg, EQ-5D or 36-item Short Form Health Survey [SF-36]) that are transformed into utility scores multiplied by the time (in years) a participant spent with that utility [32].

#### Costs

Delta societal costs or delta health care costs (hereafter referred to as delta costs), which indicate the difference in costs between the intervention and control conditions, were the primary outcome measure. Studies with a health care perspective estimated costs per study group by measuring health care use of participants during the intended follow-up period and multiplying that with a reference price for the used services. Studies with a societal perspective also included costs based on, for example, absence from paid and unpaid work, reduced productivity while at work (presenteeism), or domestic care for participants.

Two steps were taken to account for the cost differences. First, inflation was controlled for by recalculating all costs to the level of 2021 using consumer price indexes as reported for the countries in which each study was conducted [33]. Second, similar articles or services have different prices across countries, indicating differences in purchasing power. Purchasing Power Parities were used to transform costs according to the most recent rates from 2017 [34], effectively converting all costs to US dollars. A general indexing factor was used for Purchasing Power Parities rather than a health care–specific factor, as other costs were also involved when studies used a societal perspective.

### Data Preparation

#### Overview

All the data extraction items can be found in Item 2 of Multimedia Appendix 2. Apart from the outcome measures required for the meta-analysis, other data were extracted either for sensitivity analyses or for further exploration. The data required for the meta-analysis were (1) delta QALY, (2) variance of delta QALY, (3) delta costs, (4) variance of delta costs, and (5) covariance of delta QALY and delta costs. With these variables available for each study, it was possible to calculate the incremental net benefit (INB) and pool all INBs using the method described by Crespo et al [20]. The INB indicates gains of an intervention compared with another, expressed in monetary terms. Delta QALY and delta costs were either retrieved directly from the articles or calculated by subtracting the average QALYs or costs per patient in the intervention condition from those in the control condition. The method to retrieve the variances and covariance was based on that of Bagepally et al [22] and is described here in order of the most ideal (ie, reliable) to the least ideal scenario. Item 3 of Multimedia Appendix 2 lists all used formulas for this meta-analysis, including the INB.

#### Calculating the Variance of Delta QALY

##### Scenario 1

Studies report the SD, SE, or 95% CI of the delta QALY. The variance can be calculated directly by formula 1 or 2. When the 95% CI is reported, an additional step is required, for which we used formula 3.

##### Scenario 2

Studies report the SD, SE, or 95% CI of QALYs for separate conditions (but not for the difference). If the SDs of separate conditions are reported, the variance of the difference between 2 conditions can be calculated using formula 4. If the SEs of separate conditions are reported, the variance can be calculated using formula 5. If 95% CIs of separate conditions are reported, the SE is first calculated using formula 3.

##### Scenario 3

Studies report only the average of separate conditions, but no measure of spread. In this case, first the corresponding author of the article was contacted to enquire about the possibility of receiving an indication of the spread (SD, SE, or 95% CI) of delta QALY or of the spread of the average QALY gain per condition. If this was not possible or if the authors did not respond, 2 further options remained to estimate the measure of spread. First, if a cost-utility plane with bootstrapped delta QALY and delta health care or societal costs was reported, the freely available software Webplot Digitizer [35] was used to reverse engineer the individual data points. The points were exported to a Microsoft Excel file, from which the SD of the delta QALY could be calculated. An example of how Webplot Digitizer was used is described in Item 4 of Multimedia Appendix 2. Webplot Digitizer has been used in various studies and is found to be a reliable tool for extracting data with high intercoder reliability and validity [36,37]. However, the precision of the software seems to be dependent on the visual presentation of individual graphs [38]. Second, if no cost-effectiveness plane was reported, the measure of spread of delta QALY was estimated by taking the mean of the 2 most similar studies or comparisons in terms of delta QALY, number of participants, and investigated interventions.

#### Calculating the Variance of Delta Costs

For these calculations, identical steps were followed as for the variance of delta QALY but using costs instead of QALYs.

#### Calculating the Covariance Between Delta QALY and Delta Costs

##### Overview

To estimate the covariance between delta QALY and delta costs, a bootstrap procedure is necessary to be able to have multiple estimates of delta QALY and delta costs. The covariance was never reported in the included articles. As shown by Bagepally et al [22], an approximation without the original data is possible, albeit less reliable. Hence, all corresponding authors of the included studies were contacted and asked to provide the covariance to rely on author data for estimates of this parameter as much as possible.

##### Scenario 1

The authors were able to provide the covariance or information needed to calculate it; for example, the data set including all bootstrapped delta QALYs and delta costs or individual participant data needed to perform a bootstrap procedure, including (1) the allocated condition of participants, (2) the QALYs over the entire follow-up period, and (3) the costs over the entire follow-up period.

##### Scenario 2

If the authors were not able to provide the covariance or information needed to calculate it, but a cost-utility plane was presented in the article, Webplot Digitizer was used to estimate the delta QALYs and delta costs from the cost-utility plane. Finally, these data points were used to calculate the covariance.

##### Scenario 3

If the authors were not able to provide the covariance or information needed to calculate it and no cost-utility plane was presented in the article, a different approach was used. First, the mean correlation between delta QALY and delta costs was calculated for all studies, where the covariance was obtained with data received directly from the authors (ie, covariances obtained from scenario 1) using formula 6. Second, the mean correlation was used to calculate the covariance between the delta QALY and delta costs for studies falling under scenario 3 using formula 6 (mean imputation).

### Statistical Analyses

#### INB Calculations

Data preparation was performed in Excel, and analyses were conducted using Comprehensive Meta-Analysis software (version 3) [39]. First, we calculated the INB for each study. The first step is to multiply society’s willingness to pay (WTP) for 1 extra year lived in perfect health (ie, 1 QALY) with the difference in effectiveness (delta QALYs) between 2 interventions (internet intervention vs control). This expresses the difference in the effects in monetary terms. Subtracting the difference in costs between the 2 conditions (delta costs) results in INB (formula 7 in Item 3 of Multimedia Appendix 2). WTP for 1 QALY was set at US $40,000. This value was based on WTP values in high-income countries, which are typically around US $40,000 per QALY [40].

#### Pooling INBs

As studies were expected to be heterogeneous concerning follow-up periods, including costs and sampled population, a random effects approach was maintained throughout the analyses, regardless of heterogeneity scores such as the Cochran Q and *I^2^*. In accordance with this approach, study weights were corrected using the DerSimonian and Laird [41] method.

#### Moderators

Several moderators were incorporated into the analyses to explore their influence on the overall cost-effectiveness. Specifically, subgroups were based on (1) the perspective of the economic evaluation (health care vs societal), (2) length of follow-up (≥12 months vs <12 months), and (3) targeted mental disorder. Other considered subgroups were based on (4) presence of guidance (yes or no), (5) intensity of the guidance (self-guided, less than weekly, weekly, or more than weekly), (6) type of guidance (asynchronous or delayed such as email, synchronous or immediate such as chat or telephone or a combination of both), (7) method of recruitment (open or mass media or clinical referral), (8) method of diagnosis for inclusion (formal diagnosis or self-reported symptoms), (9) duration of the intervention (4-8 weeks, 9-12 weeks, >12 weeks, or undefined or unlimited access), and (10) type of control condition (care-as-usual or attention control).

#### Heterogeneity and Publication Bias

To obtain an impression of the heterogeneity between studies, *I^2^* and Cochran Q (formulas 12 and 13 in Item 3 of Multimedia Appendix 2) were calculated and reported for all analyses. However, visual inspection of the forest plot and consideration of the study characteristics were leading in the identification of between-study heterogeneity. Indications of publication bias were explored with both a visual inspection of the funnel plot and the Egger test.

#### Sensitivity Analyses

Robustness of the results was inspected in 5 sensitivity analyses. Specifically, the pooled INB was calculated separately for studies with (1) high-quality economic evaluations based on the CHEC list and (2) low risk of bias based on the Cochrane Risk of Bias tool. Two analyses—(3) and (4)—explored the impact of the value of the WTP per QALY (set at US $40,000 in the main analysis) by repeating the analysis with WTP values of US $20,000 and US $80,000, respectively. In the last analysis (5), only studies were pooled for which the covariance could be calculated directly from author data.

## Results

### Study Selection

**Figure 1:**
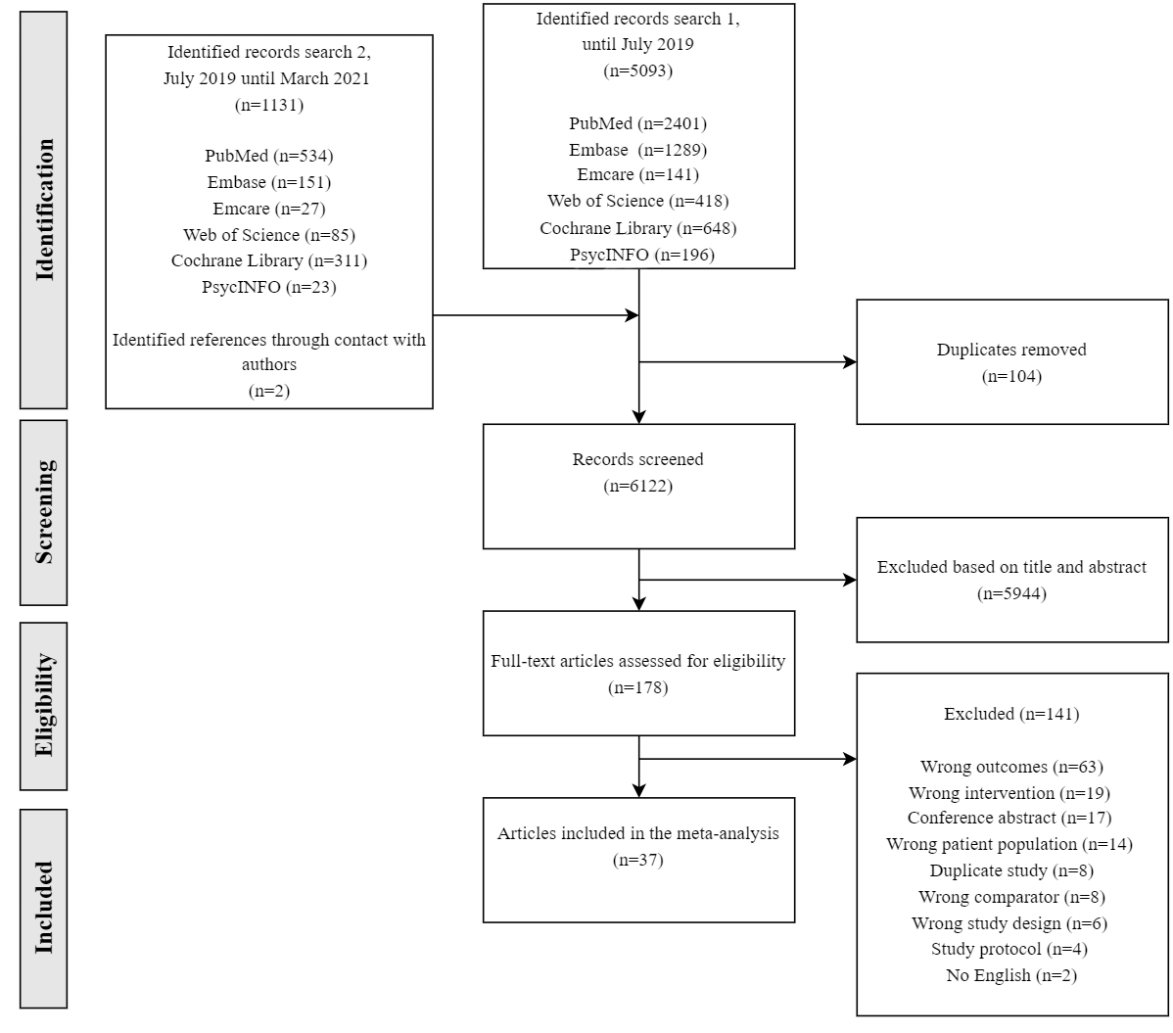
Study selection flow.

Figure 1 illustrates the study selection flow. Tables 1 and 2 present a detailed overview of all the included studies [42-77] and their outcomes. In addition, an overview of all studies that were excluded after the full-text screening phase and the reason for exclusion can be found in Item 5 of Multimedia Appendix 2. A total of 6226 papers, conference abstracts, and trial registrations were identified. Full texts of 178 papers were examined, and data from 37 articles published between 1990 and March 2021 were extracted. From 200 relevant protocols, conference abstracts, and trial registrations, follow-up was needed for 93 records with 76 different corresponding authors. For those not needing follow-up, it was clear that data gathering was still ongoing or that data were already published and included in the screening. The 76 corresponding authors were approached via email to clarify whether the data were already available to implement in our meta-analysis. One reminder was sent within 2 weeks if there was no response. This yielded 4 additional articles from 3 different authors. In total, 70% (53/76) of authors responded, and for 94% (50/53) of those responses, cost-utility data were not yet available or authors were not willing to share data at this time.

**Table 1:** Characteristics of included studies.

Characteristic		Sample, n	Study
**Country**			
	United Kingdom	12	Bogosian et al [43], Crombie et al [45], Deluca et al [47] (high risk), Deluca et al [47] (low risk), Dixon et al [48], Duarte et al [49], Hollinghurst et al [53], Lovell et al [64], Morriss et al [66], Powell et al [69], Richards et al [70], and Wright et al [76]
	Netherlands	8	Aardoom et al [42], Ferwerda et al [50], Geraedts et al [51], Gerhards et al [52], Kolovos et al [59], Lokman et al [63], van Luenen et al [74], and Warmerdam et al [75]
	Sweden	5	Holst et al [54], Jolstedt et al [57], Kraepelien et al [60], Lenhard et al [61], and Lindsäter et al [62]
	Germany	4	Buntrock et al [44], Kählke et al [58], Nobis et al [68], and Röhr et al [71]
	Australia	3	Dear et al [46], Moayeri et al [65], and Titov et al [73]
	United States	2	Joesch et al [56] and Murphy et al [67]
	Canada	1	Yan et al [77]
	Italy	1	Hunter et al [55]
	Spain	1	Romero-Sanchiz et al [72]
**Targeted disorder**			
	Depression	16	Buntrock et al [44], Dixon et al [48], Duarte et al [49], Geraedts et al [51], Gerhards et al [52], Hollinghurst et al [53], Holst et al [54], Kolovos et al [59], Kraepelien et al [60], Nobis et al [68], Romero-Sanchiz et al [72], Titov et al [73], van Luenen et al [74], Warmerdam et al [75], Wright et al [76], and Yan et al [77]
	Anxiety	7	Dear et al [46], Joesch et al [56], Jolstedt et al [57], Kählke et al [58], Lindsäter et al [62], Morriss et al [66], and Powell et al [69]
	Substance abuse	5	Crombie et al [45], Deluca et al [47] (high risk), Deluca et al [47] (low risk), Hunter et al [55], and Murphy et al [67]
	Depression or anxiety	5	Bogosian et al [43], Ferwerda et al [50], Lokman et al [63], Moayeri et al [65], and Richards et al [70]
	Obsessive compulsive disorder	2	Lenhard et al [61] and Lovell et al [64]
	PTSD^a^	1	Röhr et al [71]
	Eating disorders	1	Aardoom et al [42]
**Method of diagnosis for inclusion**			
	Formal diagnosis	13	Buntrock et al [44], Dear et al^b^ [46], Dixon et al^b^ [48], Hollinghurst et al [53], Holst et al^b^ [54], Joesch et al [56], Jolstedt et al [57], Kolovos et al [59], Lenhard et al^b^ [61], Lindsäter et al [62], Lovell et al [64], Morriss et al [66], and Romero-Sanchiz et al [72]
	Self-reported symptoms	23	Aardoom et al [42], Bogosian et al [43], Crombie et al [45], Deluca et al [47] (high risk), Deluca et al [47] (low risk), Duarte et al [49], Ferwerda et al [50], Geraedts et al [51], Gerhards et al [52], Hunter et al [55], Kählke et al [58], Kraepelien et al [60], Lokman et al [63], Moayeri et al [65], Murphy et al [67], Nobis et al [68], Powell et al [69], Richards et al^b^ [70], Röhr et al [71], Titov et al^b^ [73], van Luenen et al [74], Warmerdam et al [75], and Wright et al [76]
	Other	1	Yan et al [77] (no assessment before inclusion)
**Recruitment type**			
	Via clinical institution	20	Deluca et al [47] (high risk), Deluca et al [47] (low risk), Dixon et al [48], Duarte et al [49], Ferwerda et al [50], Hollinghurst et al [53], Holst et al [54], Hunter et al [55], Joesch et al [56], Kolovos et al [59], Kraepelien et al [60], Lovell et al [64], Moayeri et al [65], Morriss et al [66], Murphy et al [67], Richards et al [70], Romero-Sanchiz et al [72], van Luenen et al [74], Wright et al [76], and Yan et al [77]
	Open or mass media recruitment	13	Aardoom et al [42], Bogosian et al [43], Buntrock et al [44], Dear et al [46], Gerhards et al [52], Jolstedt et al [57], Kählke et al [58], Lindsäter et al [62], Nobis et al [68], Powell et al [69], Röhr et al [71], Titov et al [73], and Warmerdam et al [75]
	Other^c^	4	Crombie et al [45], Geraedts et al [51], Lenhard et al [61], and Lokman et al [63]
**Economic perspective**			
	Societal	22	Aardoom et al [42], Buntrock et al [44], Crombie et al [45], Deluca et al [47] (high risk), Deluca et al [47] (low risk), Dixon et al [48], Ferwerda et al [50], Geraedts et al [51], Gerhards et al [52], Holst et al [54], Jolstedt et al [57], Kählke et al [58], Kolovos et al [59], Kraepelien et al [60], Lenhard et al [61], Lindsäter et al [62], Lokman et al [63], Lovell et al [64], Nobis et al [68], Romero-Sanchiz et al [72], van Luenen et al [74], and Warmerdam et al [75]
	Health care	15	Bogosian et al [43], Dear et al [46], Duarte et al [49], Hollinghurst et al [53], Hunter et al [55], Joesch et al [56], Moayeri et al [65], Morriss et al [66], Murphy et al [67], Powell et al [69], Richards et al [70], Röhr et al [71], Titov et al [73], Wright et al [76], and Yan et al [77]
**Follow-up period**			
	8-12 weeks	6	Dear et al [46], Jolstedt et al [57], Moayeri et al [65], Lenhard et al [61], Lindsäter et al [62], and Warmerdam et al [75]
	4-6 months	6	Aardoom et al [42], Bogosian et al [43], Kählke et al [58], Nobis et al [68], Röhr et al [71], and van Luenen et al [74]
	8-9 months	2	Hollinghurst et al [53], Murphy et al [67]
	12-14 months	20	Buntrock et al [44], Crombie et al [45], Deluca et al [47] (high risk), Deluca et al [47] (low risk), Dixon et al [48], Geraedts et al [51], Gerhards et al [52], Holst et al [54], Hunter et al [55], Kolovos et al [59], Kraepelien et al [60], Lokman et al [63], Lovell et al [64], Morriss et al [66], Powell et al [69], Richards et al [70], Romero-Sanchiz et al [72], Titov et al [73], Wright et al [76], and Yan et al [77]
	≥18 months	3	Duarte et al [49], Ferwerda et al [50], and Joesch et al [56]
**Intervention duration**			
	<6 weeks	2	Kolovos et al [59] and Röhr et al [71]
	6-8 weeks	15	Aardoom et al [42], Bogosian et al [43], Buntrock et al [44], Dear et al [46], Duarte et al [49], Geraedts et al [51], Gerhards et al [52], Kählke et al [58], Nobis et al [68], Powell et al [69], Richards et al [70], Titov et al [73], van Luenen et al [74], Warmerdam et al [75], and Wright et al [76]
	9-12 weeks	13	Crombie et al [45], Holst et al [54], Joesch et al [56], Jolstedt et al [57], Kraepelien et al [60], Lenhard et al [61], Lindsäter et al [62], Lovell et al [64], Moayeri et al [65], Morriss et al [66], Murphy et al [67], Romero-Sanchiz et al [72], and Yan et al [77]
	>12 weeks	3	Dixon et al [48], Ferwerda et al [50], and Hollinghurst et al [53]
	Undefined or unlimited access	4	Deluca et al [47] (high risk), Deluca et al [47] (low risk), Hunter et al [55], and Lokman et al [63]
**Human guidance available**			
	Yes	27	Aardoom et al [42], Bogosian et al [43], Buntrock et al [44], Dear et al [46], Dixon et al [48], Duarte et al [49], Duarte et al [50], Ferwerda et al [50], Geraedts et al [51], Hollinghurst et al [53], Holst et al [54], Joesch et al [56], Jolstedt et al [57], Kählke et al [58], Kolovos et al [59], Kraepelien et al [60], Lenhard et al [61], Lindsäter et al [62], Lovell et al [64], Moayeri et al [65], Morriss et al [66], Murphy et al [67], Nobis et al [68], Richards et al [70], Titov et al [73], van Luenen et al [74], Warmerdam et al [75], and Wright et al [76]
	No	10	Crombie et al [45], Deluca et al [47] (high risk), Deluca et al [47] (low risk), Gerhards et al [52], Hunter et al [55], Lokman et al [63], Powell et al [69], Röhr et al [71], Romero-Sanchiz et al [72], and Yan et al [77]
**Guidance type**			
	Self-guided	10	Crombie et al [45], Deluca et al [47] (high risk), Deluca et al [47] (low risk), Gerhards et al [52], Hunter et al [55], Lokman et al [63], Powell et al [69], Röhr et al [71], Romero-Sanchiz et al [72], and Yan et al [77]
	Email or written support	11	Buntrock et al [44], Ferwerda et al [50], Geraedts et al [51], Jolstedt et al [57], Kählke et al [58], Kolovos et al [59], Kraepelien et al [60], Lindsäter et al [62], Nobis et al [68], Richards et al [70], and Warmerdam et al [75]
	Chat support	1	Hollinghurst et al [53]
	Telephone support	5	Dixon et al [48], Duarte et al [49], Lovell et al [64], Moayeri et al [65], and van Luenen et al [74]
	Videoconferencing	1	Bogosian et al [43]
	Face-to-face support	3	Joesch et al [56], Murphy et al [67], and Wright et al [76]
	Combination	6	Aardoom et al [42], Dear et al [46], Holst et al [54], Lenhard et al [61], Morriss et al [66], and Titov et al [73]
**Guidance frequency**			
	Not applicable	10	Crombie et al [45], Deluca et al [47] (high risk), Deluca et al [47] (low risk), Gerhards et al [52], Hunter et al [55], Lokman et al [63], Powell et al [69], Röhr et al [71], Romero-Sanchiz et al [72], and Yan et al [77]
	Less than weekly	3	Dixon et al [48], Hollinghurst et al [53], and Lovell et al [64]
	Weekly	21	Aardoom et al [42], Bogosian et al [43], Buntrock et al [44], Dear et al [46], Duarte et al [49], Ferwerda et al [50], Geraedts et al [51], Holst et al [54], Joesch et al [56], Jolstedt et al [57], Kählke et al [58], Kolovos et al [59], Lindsäter et al [62], Moayeri et al [65], Morriss et al [66], Nobis et al [68], Richards et al [70], Titov et al [73], van Luenen et al [74], Warmerdam et al [75], and Wright et al [76]
	More than weekly	3	Kraepelien et al [60], Lenhard et al [61], and Murphy et al [67]
**Control condition type**			
	Care-as-usual^d^	32	Aardoom et al [42], Bogosian et al [43], Buntrock et al [44], Dear et al [46], Deluca et al [47] (high risk), Deluca et al [47] (low risk), Dixon et al [48], Duarte et al [49], Ferwerda et al [50], Geraedts et al [51], Gerhards et al [52], Hollinghurst et al [53], Holst et al [54], Hunter et al [55], Joesch et al [56], Kählke et al [58], Kolovos et al [59], Kraepelien et al [60], Lenhard et al [61], Lindsäter et al [62], Lokman et al [63], Lovell et al [64], Morriss et al [66], Murphy et al [67], Nobis et al [68], Powell et al [69], Richards et al [70], Röhr et al [71], Romero-Sanchiz et al [72], Titov et al [73], Warmerdam et al [75], and Yan et al [77]
	Attention control	5	Crombie et al [45], Jolstedt et al [57], Moayeri et al [65], van Luenen et al [74], and Wright et al [76]
**Mode of delivery**			
	Website^e^	28	Aardoom et al [42], Buntrock et al [44], Dear et al [46], Duarte et al [49], Ferwerda et al [50], Geraedts et al [51], Gerhards et al [52], Holst et al [54], Hunter et al [55], Joesch et al [56], Jolstedt et al [57], Kählke et al [58], Kolovos et al [59], Kraepelien et al [60], Lenhard et al [61], Lindsäter et al [62], Lokman et al [63], Lovell et al [64], Murphy et al [67], Nobis et al [68], Powell et al [69], Richards et al [70], Romero-Sanchiz et al [72], Titov et al [73], van Luenen et al [74], Warmerdam et al [75], Wright et al [76], and Yan et al [77]
	Telephone or videoconferencing	4	Bogosian et al [43], Dixon et al [48], Moayeri et al [65], and Morriss et al [66]
	Chat	1	Hollinghurst et al [53]
	Text messaging	1	Crombie et al [45]
	App	1	Röhr et al [71]
	Game (app or website)	2	Deluca et al [47] (high risk) and Deluca et al [47] (low risk)

^a^PTSD: posttraumatic stress disorder.

^b^These studies had inclusion criteria based on both (the absence of) a formal diagnosis and self-reported symptoms or a formal diagnosis was conducted after a self-reported symptom-based inclusion.

^c^Could involve a mixture of recruitment strategies, targeting a specific group of people, or recruitment via companies.

^d^Could involve a waitlist or do-nothing approach (where participants were often allowed to use other forms of treatment during the study period) or a one-time informational session or flyer.

^e^Interventions consisted of (often weekly) modules with (cognitive-behavioral) exercises (n=27) or web-based self-monitoring (n=1).

**Table 2:** Sample and outcomes of included studies.

Study	Sample size, n	Age (years)	Female (%)	Delta QALY^a^ (SE)	Delta costs in US $ (SE)	INB^b^ in US $ (variance)
Aardoom et al [42]	354	24.2 (7.7)^c^	99	<.01 (.01)	−660 (433)	668 (489,207)
Bogosian et al [43]	40	52.7 (9.5)^c^	55	−.01 (.02)	−3216 (2056)	2976 (4,688,465)
Buntrock et al [44]	406	45.0 (11.9)^c^	73.9	.01 (.02)	169 (179)	232 (884,732)
Crombie et al [45]	825	35.0 (missing)^c^	0	−.01 (.02)	488 (357)	−728 (657,104)
Dear et al [46]	70	65.5 (5.13)^c^	60	.01 (.03)	61 (19)	339 (1,847,789)
Deluca et al [47] (high risk)	756	16.1 (0.9)^c^	50.2	−.01 (.01)	547 (722)	−930 (737,655)
Deluca et al [47] (low risk)	883	15.2 (1.0)^c^	51.7	−.01 (.01)	639 (668)	−1058 (693,923)
Dixon et al [48]	609	49.6 (12.8)^c^	68.5	<.01 (.01)	2805 (144)	−196 (281,174)
Duarte et al [49]	691	39.9 (12.7)^c^	67	−.04 (.04)	171 (145)	−1911 (2,622,289)
Ferwerda et al [50]	133	56.4 (10.0)^c^	64.9	.06 (.03)	5035 (3112)	−2675 (10,740,997)
Geraedts et al [51]	231	43.4 (9.2)^c^	62.3	<.01 (.03)	−889 (2962)	889 (10,926,230)
Gerhards et al [52]	303	44.9 (11.6)^c^	43.2	−.01 (.02)	66 (1901)	−466 (4,748,077)
Hollinghurst et al [53]	297	34.9 (11.6)^c^	68	.03 (.02)	778 (106)	302 (730,265)
Holst et al [54]	90	38.6 (11.7)^c^	77.8	−.05 (.03)	−41 (58)	−1718 (6,932,833)
Hunter et al [55]	763	49 (35-61)^d^	38.5	<.01 (<.01)	3 (4)	21 (36,931)
Joesch et al [56]	690	45.1 (13.2)^c^	71.7	.05 (.04)	257 (523)	1743 (3,150,182)
Jolstedt et al [57]	131	10.0 (1.3)^c^	53.4	<.01 (.09)	−347 (6)	42 (15,261,387)
Kählke et al [58]	264	43.3 (10.2)^c^	73.1	.01 (.00)	−374 (690)	670 (706,242)
Kolovos et al [59]	269	38.0 (11.4)^c^	53.9	.03 (0.15)	571 (310)	138 (37,849,735)
Kraepelien et al [60]	945	43.0 (12.2)^c^	72.9	.01 (.03)	−46 (496)	−1519 (4,559,865)
Lenhard et al [61]	67	14.6 (1.7)^c^	46	<.01 (<.01)	19 (5)	121 (269,039)
Lindsäter et al [62]	100	46.2 (8.8)^c^	85	<.01 (.02)	−71 (317)	243 (1,068,351)
Lokman et al [63]	220	44.2 (9.9)^c^	59.1	.02 (.02)	−5000 (2740)	5840 (9,751,347)
Lovell et al [64]	473	33 (18-77)^e^	60.3	.01 (.01)	−25 (251)	465 (430,364)
Moayeri et al [65]	110	68.1 (8.8)^c^	65.5	−.01 (.01)	−270 (23)	−54 (197,204)
Morriss et al [66]	156	32 (19-82)^e^	69.2	.07 (.07)	−1419 (1299)	4219 (23,565,882)
Murphy et al [67]	507	34.9 (10.9)^c^	37.9	<.01 (.01)	147 (200)	−187 (170,067)
Nobis et al [68]	256	51.0 (12.0)^c^	62.9	.01 (.01)	1001 (999)	278 (1,457,706)
Powell et al [69]	2116	37.2 (13.8)^c^	80.2	.01 (.01)	−87 (82)	647 (51,304)
Richards et al [70]	361	29 (18)^d^	71.5	.02 (.01)	125 (65)	707 (292,998)
Röhr et al [71]	133	33.3 (11.2)^c^	38.3	<.01 (.01)	−124 (139)	−36 (64,494)
Romero-Sanchiz et al [72]	296	42.9 (10.3)^c^	75.7	.08 (.03)	−265 (459)	3526 (1,329,167)
Titov et al [73]	54	65.3 (3.0)^c^	70.4	.01 (<.01)	33 (25)	447 (30,236)
van Luenen et al [74]	188	46.3 (10.6)^c^	11.7	.01 (<.01)	13 (171)	1180 (690,364)
Warmerdam et al [75]	263	45.0 (12.1)^c^	71.1	.01 (.01)	276 (613)	124 (442,031)
Wright et al [76]	139	15.0 (1.4)^c^	64	.03 (.06)	−20 (161)	1180 (5,895,741)
Yan et al [77]	1407	47.1 (17.0)^c^	73	.01 (.01)	−140 (36)	356 (1491)

^a^QALY: quality-adjusted life-year.

^b^INB: incremental net benefit.

^c^Mean (SD).

^d^Median (IQR).

^e^Median (range).

### Data Preparation

For 7 studies, no measure of spread of delta QALY or delta costs could, directly or indirectly, be deduced from the article. Data from 1 study were available within our research team [42]. After enquiring with the authors of the other 6 studies (5 of which responded), data of only 3 comparisons were still missing. Two were solved using Webplot Digitizer on the presented cost-effectiveness plane, and the other was solved by mean imputation based on 2 comparisons within the same study.

All authors of the included studies were contacted to provide information on the covariance between delta QALY and delta costs. Authors responded for 84% (31/37) of the included studies; however, not all authors were able to provide information on the covariance. Specifically, for 12 of the studies, the covariance was based on data from the authors. The mean Pearson correlation (*r*) between delta QALY and delta costs based on these 12 studies was −0.12 (SD 0.16). For the remaining 25 studies, covariances were calculated using Webplot Digitizer (n=13) or using the estimated mean correlation calculated earlier (n=12).

### Characteristics of Included Studies

In total, the 37 included studies (Table 1) recruited 15,596 participants, ranging between 40 and 2116. Some study conditions were irrelevant for this meta-analysis (eg, an active intervention without an internet component), so the main analysis was based on 14,946 participants. Intention-to-treat analyses were conducted in most of the studies (32/37, 86%). Mental disorders that were targeted were depression (16/37, 43%), anxiety (7/37, 19%), alcohol or substance abuse (5/37, 13%), depression and anxiety simultaneously (5/37, 13%), obsessive compulsive disorder (2/37, 5%), posttraumatic stress disorder (1/37, 3%), and eating disorders (1/37, 3%). Experimental interventions were mostly cognitive behavior–based modules or websites that participants could engage with (29/37, 78%). Other interventions consisted of teleconferencing (2/37, 5%), chat or SMS text messaging (2/37, 5%), a web-based game (2/37, 5%), and telephone support (2/37, 5%). Some form of guidance within the intervention was available in 27 studies, whereas the intervention was self-guided in 10 studies. Guidance consisted of written feedback (11/27, 41%), telephone calls (5/27, 18%), face-to-face communication, including teleconferencing (4/27, 15%), chat (1/27, 4%), or a combination of these (6/27, 22%). Control conditions of the studies included waiting list or care-as-usual conditions (32/37, 86%) and psychological placebo or attention control conditions (5/37, 13%). The follow-up periods ranged from 8 weeks to 2 years, with most studies (20/37, 54%) maintaining a 12-month follow-up period.

### Quality of Life

Questionnaires used to calculate QALYs were EQ-5D (32/37, 86%), KIDSCREEN-10 (1/37, 3%), SF-6D or SF-36 (3/37, 8%), and Australian quality of life instrument (1/37, 3%). The pooled difference in effectiveness (intervention QALY gains minus control QALY gains) for the included studies was 0.004 QALY (SE 0.002; Hedges *g*=0.052, 95% CI 0.010-0.094; *P*=.02). Although the difference was statistically significant, likely because of the large sample size, the size of the difference was deemed negligible.

### Costs

Main questionnaires used to measure health care use and costs in the included studies were the Treatment Inventory of Costs in Psychiatric Patients (n=14) and Client Service Receipt Inventory (n=7), but other or self-administered questionnaires, medical records, and diaries were also used. In total, 15 studies reported costs from a health care perspective and 22 presented a societal perspective. The pooled difference in costs (intervention costs minus control costs) when studies with a health care and societal perspective were taken together was US $49 (SE 40; Hedges *g*=0.002, 95% CI −0.080 to 0.84; *P*=.96). Considering the uncertainty in measurements of costs, the small difference indicates that internet interventions were equally expensive as control conditions. Results for studies with different economic perspectives were similar, with no difference in costs for studies with a health care (US $40, SE 44; Hedges *g*=−0.026, 95% CI −0.21 to 0.16; *P*=.78), and societal perspective (US $158, SE 76; Hedges *g*=0.034, 95% CI −0.012 to 0.080; *P*=.15).

### Cost-effectiveness

**Figure 2:**
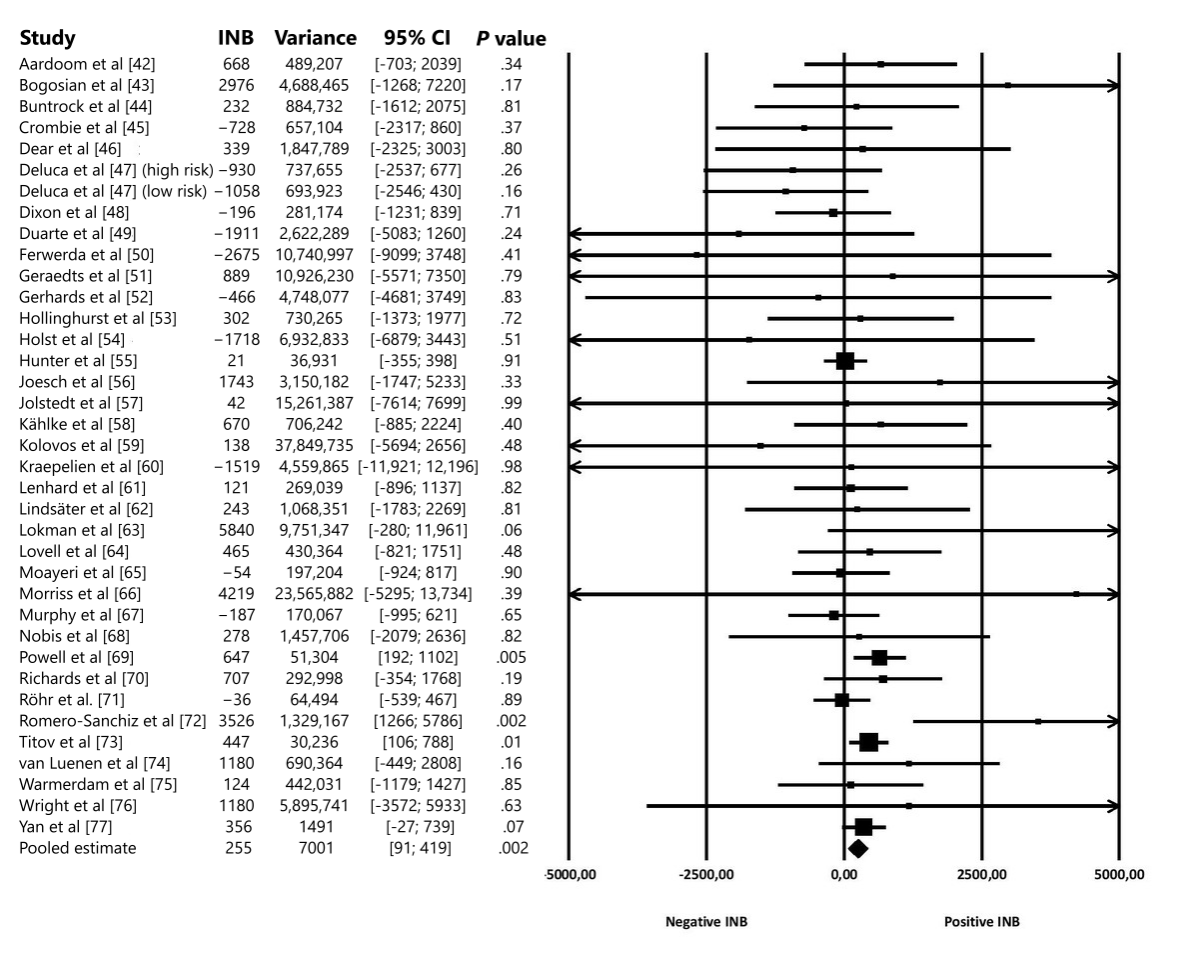
Forest plot of incremental net benefits (INBs) and the pooled estimate according to a random effects model.

Visual inspection of individual INBs and their 95% CIs (Figure 2) indicated substantial heterogeneity among the 37 included studies. Statistical measures of heterogeneity suggested otherwise (Cochran *Q*_36_=37.12, *P*=.42; *I*^2^=3.0%, 95% CI 0.0%-42.8%) but are difficult to interpret because of the large 95% CI of *I^2^* and considerable within-study uncertainty. In other words, the between-study heterogeneity seemed to be overshadowed by the large within-study heterogeneity. Therefore, a random effects model was preferred over a fixed effects model to pool INBs. The INB was positive (more favorable balance of costs and effects in the internet intervention compared with the control condition) in 25 of the included RCTs. Furthermore, at a WTP of US $40,000 per QALY, the pooled INB was US $255 (95% CI US $91 to US $419; *P*=.002). The results suggest that internet interventions are slightly more cost-effective compared with a do-nothing or care-as-usual approach.

### Moderator Analyses

Pooled INB values were also calculated for the subgroups based on the 10 moderator variables. Outcomes for subgroups based on perspective, length of follow-up, and targeted mental disorder are presented in the text, and results for all moderator (including presence of guidance, intensity of guidance, type of guidance, recruitment strategy, diagnosis for inclusion, intervention duration, and control condition type) analyses are presented in Item 6 of Multimedia Appendix 2.

Pooling studies from an economic perspective influenced the results. Specifically, looking at studies with a health care perspective separately (n=15), the pooled INB was US $280 (95% CI US $109 to US $451; *P*=.001). For studies with a societal perspective (n=22), the pooled INB was substantially lower at US $161 (95% CI US $247 to US $569; *P*=.44). This suggests that the cost-effectiveness of internet interventions compared with control conditions cannot be assumed when maintaining a societal perspective.

Studies with a short (<12 months) follow-up (n=14) had a pooled INB of US $112 (95% CI US $194 to US $418; *P*=.47) and studies with a long (≥12 months) follow-up (n=23) had a pooled INB of US $270 (95% CI US $14 to US $554; *P*=.06). For RCTs with a long follow-up period, statistical significance was likely not attained because the sample size decreased compared with the main analysis. Nevertheless, the studies with a long follow-up, which are usually better able to capture all relevant costs and effects than those with a short time horizon [78,79], had a pooled estimate comparable with the estimate of all studies taken together. This strengthens the idea that internet interventions are likely to be cost-effective compared with control conditions in the long term.

Concerning targeted mental disorders, a significant positive INB was found for internet interventions targeting anxiety (n=7; US $644, 95% CI US $227 to US $1062; *P*=.002) and depression (n=16; US $387, 95% CI US $156 to US $618, *P*=.001). Similarly, the INB of studies with internet interventions targeting depression and anxiety simultaneously (n=5), pooled INB of US $580 (95% CI US $584 to US $1744; *P*=.33), and obsessive compulsive disorder (n=2), pooled INB of US $253 (95% CI US $544 to US $1051; *P*=.53), was positive. The size of the INB for these 2 groups also indicated that cost-effectiveness compared with control conditions was likely, but statistical significance was not attained. Internet interventions were unlikely to be cost-effective when targeting alcohol or substance abuse (n=5), with a pooled INB of US $129 (95% CI US $448 to US $191; *P*=.43). It must be noted that of the 2 studies regarding obsessive compulsive disorder, one targeted children, further obscuring the interpretability for this subgroup. Only 1 study was available for eating disorders and posttraumatic stress disorder, rendering it impossible to pool.

### Risk of Bias and Quality of Economic Evaluation

Table 3 presents details on the risk of bias, and Table 4 presents details on the quality ratings of the economic analysis for the included studies. The overall risk of bias of the included RCTs was considered high, with a low risk of bias for 8, medium risk of bias for 8, and high risk of bias for 21 studies. The economic appraisal of the included studies, based on the CHEC list, suggested moderate quality, with 24 studies receiving a high-quality rating and 13 receiving a low-quality rating. The level of agreement between the raters was considered low for the risk of bias assessment (55% agreement) and high for the CHEC list ratings (89% agreement).

**Table 3:** Cochrane Risk of Bias table of included studies.

Study	Random sequence generation	Allocation concealment	Blinding of participants and personnel	Incomplete outcome data	Blinding of outcome assessors	Selective reporting	Other bias	Overall risk of bias
Aardoom et al [42]	+^a^	+	−^b^	+	+	+	+	Low
Bogosian et al [43]	+	+	−	+	+	?^c^	+	Low
Buntrock et al [44]	+	+	−	+	+	+	+	Low
Crombie et al [45]	+	+	+	+	+	+	+	Low
Dear et al [46]	+	+	−	−	?	?	+	High
Deluca et al [47] (high risk)	+	+	−	+	−	+	+	High
Deluca et al [47] (low risk)	+	+	−	+	−	+	+	High
Dixon et al [48]	+	+	−	+	+	+	?	Medium
Duarte et al [49]	+	+	−	+	?	+	−	High
Ferwerda et al [50]	+	+	−	+	−	?	+	High
Geraedts et al [51]	+	+	−	+	+	?	+	Low
Gerhards et al [52]	+	+	−	+	−	?	+	High
Hollinghurst et al [53]	+	+	−	?	−	?	−	High
Holst et al [54]	+	+	−	−	?	+	?	High
Hunter et al [55]	+	+	−	−	+	+	−	High
Joesch et al [56]	+	+	−	+	−	+	+	High
Jolstedt et al [57]	+	+	−	+	+	+	+	Low
Kolovos et al [59]	+	+	−	?	+	+	+	Medium
Kählke et al [58]	+	?	−	+	+	+	+	Medium
Kraepelien et al [60]	+	+	−	+	+	?	+	Low
Lenhard et al [61]	+	+	−	+	+	+	−	High
Lindsäter et al [62	+	+	−	+	+	+	+	Low
Lokman et al [63]	?	+	−	+	−	?	−	High
Lovell et al [64]	+	+	−	?	+	+	−	High
Moayeri et al [65]	+	+	−	+	+	?	?	Medium
Morriss et al [66]	+	+	−	+	+	?	?	Medium
Murphy et al [67	+	+	−	?	+	?	−	High
Nobis et al [68]	+	+	−	+	+	−	+	High
Powell et al [69]	+	+	−	+	−	?	+	High
Richards et al [70]	+	−	−	−	−	+	+	High
Röhr et al [71]	+	+	−	?	+	+	+	Medium
Romero-Sanchiz et al [72]	+	+	−	+	+	?	?	Medium
Titov et al [73]	+	+	−	−	?	?	?	High
van Luenen et al [76]	+	+	−	?	+	?	+	Medium
Warmerdam et al [75]	+	?	−	+	+	?	−	High
Wright et al [76]	+	+	−	+	−	?	?	High
Yan et al [77]	−	−	−	?	−	+	−	High

^a^Item scored as low risk of bias.

^b^Item scored as having a high risk of bias.

^c^Item scored as having an unclear risk of bias.

**Table 4:** Quality of the economic evaluation of included studies using the CHEC list.

Study	1^a^	2^b^	3^c^	4^d^	5^e^	6^f^	7^g,h^	8^h,i^	9^h,j^	10^h,k^	11^h,l^	12^h,m^	13^h,n^	14^h,o^	15^p^	16^q^	17^r^	18^s^	19^t^	Sum score	Quality
Aardoom et al [42]	+^u^	+	+	+	−^v^	+	+	+	+	+	+	+	+	+	+	+	+	+	−	17/19	High
Bogosian et al [43]	+	+	+	+	−	−	+	+	+	+	+	+	+	+	−	+	−	+	−	14/19	High
Buntrock et al [44]	+	+	+	+	+	+	+	+	+	+	+	+	+	+	+	+	+	+	+	19/19	High
Crombie et al [45]	+	+	+	+	+	+	+	+	+	+	+	−	+	+	+	+	+	+	+	18/19	Low
Dear et al [46], 2015	+	+	+	+	−	−	−	+	+	+	+	+	+	+	−	+	+	+	+	14/19	Low
Deluca et al [47] (high risk)	+	+	+	+	+	+	+	+	+	+	+	+	+	−	−	+	−	+	−	15/19	Low
Deluca et al [47] (low risk)	+	+	+	+	+	+	+	+	+	+	+	+	+	−	−	+	−	+	−	15/19	Low
Dixon et al [48]	+	+	+	+	+	+	+	+	+	+	+	+	+	+	+	+	−	+	−	17/19	High
Duarte et al [49]	+	+	+	+	+	−	+	+	+	+	+	+	+	+	+	+	+	+	−	17/19	High
Ferwerda et al [50]	+	+	+	+	+	+	+	+	+	+	+	+	+	+	+	+	−	+	−	17/19	High
Geraedts et al [51]	+	+	+	+	+	+	+	+	+	+	+	+	+	+	+	+	+	+	−	18/19	High
Gerhards et al [52]	+	+	+	+	+	+	+	+	+	+	+	+	+	+	+	+	+	+	−	18/19	High
Hollinghurst et al [53]	+	+	+	+	−	−	+	+	+	+	+	+	+	+	+	+	−	+	+	16/19	High
Holst et al [54]	+	+	+	+	+	+	+	+	−	+	+	+	+	+	+	+	−	+	−	16/19	Low
Hunter et al [55]	+	+	+	+	−	−	+	+	+	+	+	+	+	+	+	+	−	+	−	15/19	High
Joesch et al [56]	+	+	−	+	+	−	−	+	+	+	+	+	+	−	−	+	+	+	−	13/19	Low
Jolstedt et al [57]	+	+	+	+	−	+	+	+	+	+	+	+	+	+	−	+	+	+	+	17/19	High
Kählke et al [58]	+	+	+	+	−	+	+	+	+	+	+	+	+	+	+	+	+	+	+	18/19	High
Kolovos et al [59]	+	+	+	+	+	+	+	+	+	+	+	+	+	+	+	+	+	−	−	17/19	High
Kraepelien et al [60]	+	+	+	+	+	+	+	+	+	+	+	+	+	+	+	+	+	+	−	18/19	High
Lenhard et al [61]	+	+	+	+	−	+	+	+	+	+	+	+	+	+	+	+	+	+	+	18/19	High
Lindsäter et al [62]	+	+	+	+	−	+	+	+	+	+	+	+	+	+	+	+	+	+	−	17/19	High
Lokman et al [63]	+	+	−	+	+	+	+	+	+	+	+	−	−	+	+	−	+	+	−	14/19	Low
Lovell et al [64]	+	+	+	+	+	+	+	+	+	+	+	+	+	+	+	+	+	−	−	17/19	High
Moayeri et al [65]	+	+	+	+	−	−	+	+	+	+	+	+	+	+	+	+	+	+	−	16/19	High
Morriss et al [66]	+	+	+	+	+	+	+	+	+	+	+	+	+	+	−	+	+	+	−	17/19	High
Murphy et al [67]	+	+	+	+	−	+	−	+	+	+	+	+	+	+	+	+	+	+	−	16/19	Low
Nobis et al [68]	+	+	+	+	−	+	+	+	+	+	+	+	+	+	+	+	+	−	−	16/19	High
Powell et al [69]	+	+	−	+	+	+	+	−	+	+	+	+	+	+	−	+	+	+	−	14/19	Low
Richards et al [70]	+	+	+	−	+	−	+	+	+	+	−	+	+	+	+	+	+	+	−	15/19	Low
Röhr et al. [71]	+	+	+	+	−	−	+	+	+	+	+	+	−	+	+	+	+	+	−	15/19	Low
Romero-Sanchiz et al [72]	+	+	+	+	+	+	+	+	+	+	+	+	+	+	−	+	+	+	−	17/19	High
Titov et al [73]	+	+	+	+	−	−	+	+	+	+	+	+	+	+	−	+	+	+	−	15/19	High
van Luenen et al [74]	+	+	+	+	−	+	+	+	+	+	+	−	+	+	+	+	+	+	−	16/19	Low
Warmerdam et al [75]	+	+	+	+	−	+	+	+	+	+	+	+	+	+	+	+	+	+	−	17/19	High
Wright et al [76]	+	+	−	+	+	+	+	+	+	+	+	+	+	+	−	+	+	+	−	16/19	High
Yan et al [77]	+	+	+	−	+	−	+	+	−	+	−	+	+	+	+	+	−	+	−	13/19	Low

^a^Is the study population clearly described?

^b^Are competing alternatives clearly described?

^c^Is a well-defined research question posed in answerable form?

^d^Is the economic study design appropriate to the stated objective?

^e^Is the chosen time horizon appropriate to include relevant costs and consequences?

^f^Is the actual perspective chosen appropriate?

^g^Are all important and relevant costs for each alternative identified?

^h^Necessary item for a high-quality score.

^i^Are all costs measured appropriately in physical units?

^j^Are costs valued appropriately?

^k^Are all important and relevant outcomes for each alternative identified?

^l^Are all outcomes measured appropriately?

^m^Are outcomes valued appropriately?

^n^Is an incremental analysis of costs and outcomes of alternatives performed?

^o^Are all future costs and outcomes discounted appropriately?

^p^Are all important variables, whose values are uncertain, appropriately subjected to sensitivity analysis?

^q^Do the conclusions follow from the data reported?

^r^Does the study discuss the generalizability of the results to other settings and patient or client groups?

^s^Does the article indicate that there is no potential conflict of interest of study researchers and funders?

^t^Are ethical and distributional issues discussed appropriately?

^u^Item scored as sufficient or high quality.

^v^Item scored as insufficient or low quality.

### Publication Bias

**Figure 3:**
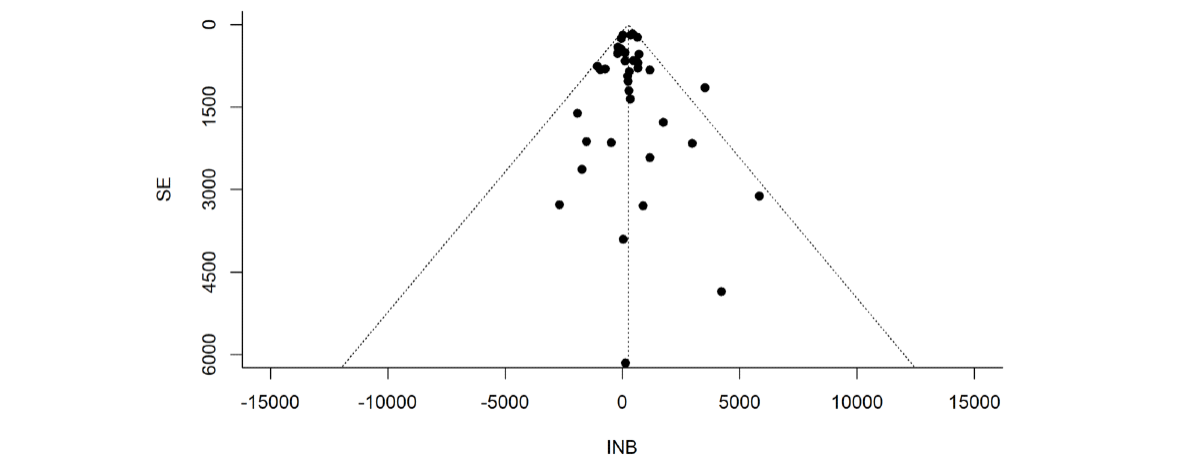
Funnel plot of standard error by incremental net benefit (INB) for inspecting publication bias.

A funnel plot of the included studies is shown in Figure 3. Visual inspection of the plot suggested some evidence for publication bias. The Egger test did not indicate asymmetry in the funnel plot (*z*=−0.026, 1-tailed *P*=.80).

### Sensitivity Analyses

Results of all sensitivity analyses are presented in Item 7 of Multimedia Appendix 2. First, the main analysis was repeated for studies with a high-quality rating on the CHEC list (n=24), resulting in a pooled INB of US $253 (95% CI US $43 to US $463; *P*=.02). This suggests cost-effectiveness of internet interventions was maintained when looking at high-quality studies alone and that results from the main analysis were not dependent on low-quality studies. Second, the pooled INB for studies with a low risk of bias (n=8) was US $244 (95% CI US $555 to US $1042; *P*=.55). A similar result was found when medium risk of bias was considered as a low risk of bias (n=16), with a pooled INB of US $216 (95% CI US $182 to US $615; *P*=.29). The pooled INB for RCTs with low risk of bias was comparable with that of the main analysis, suggesting that the pooled result of all 37 studies was not critically biased. However, for high risk of bias studies, the pooled INB was slightly higher, US $272 (95% CI US $68 to US $475; *P*=.009). This finding suggests a small overestimation in the overall pooled estimate. Third, when 1 QALY was valued at US $20,000 instead of US $40,000, the pooled INB of the 37 studies was US $145 (95% CI US $56 to US $234; *P*=.001). At a WTP of US $80,000 for 1 QALY, the pooled INB was US $431 (95% CI US $115 to US $747; *P*=.008). The 2 analyses show that if society’s WTP for 1 additional QALY for an individual is lower (US $20,000) or higher (US $80,000) than US $40,000, the cost-effectiveness of internet interventions compared with care-as-usual is still expected. Finally, the pooled INB of the studies for which the covariance could be calculated directly from author data (n=12) was US $264 (95% CI US $167 to US $694; *P*=.23). This was similar to the pooled estimate of all 37 studies, indicating that the way the covariance was calculated did not greatly influence the results of the meta-analysis.

### Deviations From the Protocol

An individual participant data meta-analysis was not achievable, so moderator analyses based on age, gender, and symptom severity were not possible or feasible. In addition, a moderator analysis with subgroups based on whether the study was conducted by developers of the interventions was planned, but this information proved too difficult to find for many of the studies.

## Discussion

### Principal Findings

This systematic review and meta-analysis aimed to research the pooled evidence of the cost-effectiveness of internet interventions for mental disorders compared with control conditions. The results indicated that internet interventions were negligibly more effective in terms of QALY gains and equally costly compared with control conditions but might be cost-effective. Internet interventions had an INB of US $255 (95% CI US $91 to US $419) compared with control conditions when society is willing to pay US $40,000 for 1 QALY improvement for an individual. INBs were still positive when WTP for 1 additional QALY was lower (US $20,000) or higher (US $80,000). The results suggest that internet interventions for mental disorders are likely to be cost-effective compared with a do-nothing approach, especially when they target depression or anxiety and when some form of guidance is added to the intervention. This is especially interesting considering that internet interventions are now frequently and successfully being implemented [80] and can be used to serve populations that need mental health care but do not yet receive it [81]. Financing such scalable and, in some cases, anonymous interventions comes with difficulties, as they often do not fit in the funding possibilities for traditional treatments. Nevertheless, findings from this meta-analysis indicate that doing so might ultimately help reduce the global burden of mental disorders.

Moderator analyses revealed that cost-effectiveness of internet interventions compared with control conditions was maintained for studies with a health care perspective but not for those with a societal perspective. An explanation could be that indirect costs included in a societal perspective are usually higher and measured with more uncertainty than direct health care costs. Indeed, studies with a health care perspective included in the analyses had both a smaller cost range and smaller pooled SE compared with studies with a societal perspective. A relatively small difference in health care costs might then be obscured by large indirect costs. This reflects a larger problem that sample size calculations for RCTs are almost exclusively based on detecting differences in disorder-specific effectiveness while neglecting QALYs and costs, rendering the trial unlikely to be adequately powered for an economic evaluation [18]. Furthermore, compared with control conditions, internet interventions were likely to be cost-effective for symptoms of anxiety, depression, and obsessive compulsive disorder. This was not the case for alcohol and substance abuse. The finding that internet interventions for alcohol and substance abuse were not found to be cost-effective compared with controls might be because of a lack of power in that subgroup. Alternatively, it could relate to the absence of guidance. Indeed, guided interventions were found to be cost-effective compared with care-as-usual, whereas self-guided interventions were not, and none of the alcohol and substance abuse studies incorporated guidance. Adding guidance to an internet intervention might have a positive effect on cost-effectiveness. However, self-guided interventions have fewer functions than guided ones and do not compensate for this by higher levels of technical sophistication (ie, responsiveness) at least for those targeting depression [82], perhaps partly explaining the positive influence of guidance in internet interventions. Another result was that studies with a follow-up period of ≥12 months generally showed internet interventions for mental disorders to be efficient compared with a control condition, while this was not the case for studies with shorter follow-up periods. It is possible that some factors that influence the efficiency of internet interventions become apparent after a certain time only. For example, effects of an intervention concerning health care visits or work productivity may take a while to attain. This substantiates the idea that follow-up periods of at least 12 months are important when conducting an economic evaluation of such interventions [78,79]. Several other findings of subgroup analyses are worth mentioning. First, recruiting participants through (social) media rather than by clinical referral was more likely to yield a positive INB. Speculatively, studies that used an open recruitment system included participants who were less severely ill or more strongly motivated for this type of intervention, thereby benefitting more. Comparing study subgroups based on the severity of symptoms, motivation to change, or related participant characteristics directly was not feasible for this meta-analysis but could be an interesting avenue for future research. Second, shorter interventions were likely to be more efficient compared with controls, whereas this was not true for longer interventions. It might be that longer internet interventions cost more but do not perform better compared with shorter interventions in terms of QALYs or health care or societal costs. Third, cost-effectiveness of internet interventions for mental disorders was not likely when control conditions included an active component (ie, attention control). Accordingly, activating participants seems to be an important component for an efficient intervention, while the content may be of lesser importance. Moderator analyses should be considered as exploratory because of the small number of studies in some subgroups.

### QALYs and Mental Health Interventions

Interestingly, internet interventions were found to be only marginally more effective than control conditions in terms of QALY gain. It is surprising that internet interventions produced practically the same amount of QALYs as a do-nothing approach, given the substantial evidence for the effectiveness of such interventions [12]. However, it is likely that the internet interventions were more effective in terms of symptom reduction and other areas of well-being, but these improvements were not captured well by the generic health-related quality-of-life measures (eg, EQ-5D and SF-36) used in economic evaluations. Such instruments capture only a selective number of domains of quality of life and use an almost exclusive focus on people’s current functional abilities with little emphasis on coping capabilities and resources [83]. Consequently, they might not be suitable in contexts outside of health, such as chronic illness, older adult care, general well-being, and mental illness [84]. Future economic analyses of internet interventions for mental disorders should consider using other instruments, such as the ICECAP-A [85], which measures well-being beyond physical health, to complement generic health questionnaires [86,87].

### Meta-analyses on Cost-effectiveness Data

To our knowledge, meta-analyses on cost-effectiveness studies have only been attempted in 5 studies in different fields of medicine by one other research team [21-25] but not yet in the area of mental health interventions. The theoretical method by Crespo et al [20] has been practically applied and explained by Bagepally et al [21]. This study built on this method by improving the precision of estimating covariances between delta costs and delta QALYs. In general, much data are necessary to pool cost-effectiveness outcomes in a meta-analysis, some of which are often not reported. Enhancing the quality of economic evaluations by clearly reporting costs, QALYs, and indicators of spread (eg, SD or SE) for all studied conditions or open availability of study data makes conducting meta-analyses on cost-effectiveness data more feasible. In addition to these practical challenges, the unavoidable and substantial heterogeneity between cost-effectiveness studies alongside RCTs might make statistically pooling outcomes undesirable [88]. Therefore, careful planning and cautious interpretation of the findings are warranted when considering a meta-analysis on cost-effectiveness data. Nevertheless, bearing the limitations in mind, several considerations corroborated the choice for pooling cost-effectiveness data. First, by adhering to clear predefined inclusion and exclusion criteria throughout the search procedure, the included studies were comparable across many characteristics. For example, the interventions were often similar in terms of content, mode of delivery, duration, and frequency. In addition, in most studies, the control conditions consisted of a do-nothing approach (ie, participants did not receive the internet intervention but were allowed to continue receiving the care they received before the study). Similarly, 21 of the included studies used the Treatment Inventory of Costs in Psychiatric Patients or Client Service Receipt Inventory to measure service use, suggesting that these studies considered the same costs, and almost all studies used the EQ-5D for calculating QALYs. Second, modeling studies were excluded to avoid additional variation between methods. Third, characteristics that were thought to have a large influence on the outcomes were tested in moderator and sensitivity analyses. Overall, these analyses were in line with the overall pooled estimate, further substantiating the robustness of the finding that internet interventions are likely to be cost-effective compared with control conditions. Finally, the variance of the INB of individual studies was often large. Consequently, a meta-analysis was important because it offered insight beyond individual studies, which are rarely powered to detect differences in QALYs and costs [18].

### Limitations

Although the main, moderator, and sensitivity analyses point in the direction of internet interventions being cost-effective compared with control conditions, the findings should be interpreted with considerable caution and are difficult to generalize beyond the current sample of included studies. The first limitation was that the studies were heterogeneous in terms of included costs and follow-up duration, which warrants careful interpretation of the overall pooled results. Second, variances of the cost-effectiveness outcomes were large to such an extent that they overshadowed the differences between studies, making it difficult to understand and quantify between-study heterogeneity. Third, publication bias possibly shifted results in favor of internet interventions because economic analyses are often a last step in effectiveness research and some results might never get published. For example, some contacted authors who published a protocol mentioning an economic analysis replied that such an analysis was ultimately not attempted, as the internet intervention was not found to be effective. To counter this problem, researchers are encouraged to designate in their RCT study protocol whether an economic analysis will be attempted and perform it regardless of the results on effectiveness. A fourth reason to interpret the results with caution is that the risk of bias in most RCTs was high, possibly leading to a slight overestimation of the efficiency of internet interventions compared with control conditions. However, when quality was assessed using the CHEC list, which is arguably more suitable in the case of economic evaluations, high-quality studies showed a positive INB, whereas low-quality studies did not. This confirms the idea that internet interventions might be cost-effective compared with controls only when the economic evaluation is of adequate quality. The subjectively chosen, though theory driven, cutoff points for the risk of bias and CHEC list should be considered when considering the importance of these sensitivity analyses. Indeed, studies designated as high quality based on the CHEC might not have had an appropriate time horizon or economic perspective.

### Implications and Future Directions

This study provides an overview of published articles on the cost-effectiveness of internet interventions for mental disorders and their findings and provides insights for future research and implementation steps. First, it must be noted that the results should be replicated in other meta-analyses before the clinical and policy implications can be reliably stated. To accomplish this, standardization of economic evaluations in the area of mental health, as has been done in other sectors [89,90], would be helpful. Consequently, included studies in similar meta-analyses would be more homogeneous and easier to compare. Nevertheless, the pooled estimates of the main and subgroup analyses complement the results of the individual studies. They suggest that policy makers, insurance companies, and subsidy providers should invest in internet interventions for mental disorders, as they appear to be an efficient way of improving quality of life compared with not offering them. For clinical practice, often facing financial pressure and waiting lists, this might involve a transition where internet interventions are increasingly embraced and become an integral part of the treatment options. The results of this meta-analysis also suggest that some components of internet interventions for mental disorders, such as recruitment strategy, symptom severity, guidance, and length of the intervention, are worth considering upon implementation. This is the first study to pool cost-effectiveness outcomes in the area of psychiatry, paving the way for other researchers to apply this method to new meta-analyses to further enhance the understanding of the cost-effectiveness of internet interventions compared with alternatives. For example, this study only considered control conditions, as internet interventions are often used for early detection and underserved populations, for which care-as-usual and a waiting list are realistic comparators. Future work could compare internet interventions with active comparators, such as face-to-face treatment, to clarify the difference in effects and costs between these forms of treatment. In addition, this study looked broadly at the topic of cost-effectiveness of internet interventions for mental disorders and should be considered as a starting point. It might be valuable to investigate a study sample with more homogeneous interventions or designs to obtain more precise estimates of cost-effectiveness. Relatedly, modeling studies were not considered in this meta-analysis, as pooling the outcomes from such studies with those obtained from RCTs was undesirable. Performing a meta-analysis on modeling studies is feasible, however, and might be an interesting extension. Importantly, no study from a non-Western culture or low-income country was included in the study, while especially low-income countries or people in areas where health care is not paid for by the government might benefit from internet interventions. Initiating internet interventions in such contexts might be a challenge but can help to reduce health care costs in the long term. Conducting research on the cost-effectiveness of internet interventions in low-income countries is therefore highly recommended.

### Conclusions

Pooling outcomes of 37 studies revealed a small benefit of internet interventions for mental disorders compared with control conditions. The perspective of the economic evaluation targeted mental disorder and WTP for 1 QALY moderated the results. Generalizability to new studies is poor given the large variance of the outcome of interest and heterogeneity between studies. The findings show that cost-effectiveness of internet interventions for mental disorders compared with a do-nothing or care-as-usual approach is likely but not guaranteed. The continuation of high-quality and adequately powered economic evaluations is necessary. This is the first study in the field of psychiatry to pool cost-effectiveness outcomes in an aggregate data meta-analysis, paving the way for other researchers to use and expand this method.

## Appendix

Multimedia Appendix 1 PRISMA (Preferred Reporting Items for Systematic Reviews and Meta-Analyses) checklist.

Multimedia Appendix 2 Supplemental materials.

## Abbreviations

CHEC: Consensus Health Economic Criteria
INB: incremental net benefit
PRISMA: Preferred Reporting Items for Systematic Reviews and Meta-Analyses
QALY: quality-adjusted life-year
RCT: randomized controlled trial
SF-36: 36-item Short Form Health Survey
WTP: willingness to pay
