# Supplementary material for: Cost-effectiveness of Internet Interventions Compared With Treatment as Usual for People With Mental Disorders: Systematic Review and Meta-analysis of Randomized Controlled Trials
Source: J Med Internet Res. 2023 Jan 5;25:e38204. doi: 10.2196/38204 (PMC9893732; doi:10.2196/38204)
Supplement: Multimedia Appendix 2 [file jmir_v25i1e38204_app2.docx]

**Cost-Effectiveness of eHealth Interventions Compared to Treatment as Usual for People with Mental Disorders: A Systematic Review and Meta-Analysis of Randomized Controlled Trials**

**Content**

[Item 1. Full Pubmed search string 2](#_Toc123716269)

[Item 2. Data extraction items 5](#_Toc123716270)

[Item 3. Formulas used for data preparation and final analyses. 8](#_Toc123716271)

[Item 4. Example of the estimation of the covariance between delta QALY and delta costs with the use of Webplot Digitizer 10](#_Toc123716272)

[Item 5. Excluded studies with reasons for exclusion 11](#_Toc123716273)

[Item 6. Results on moderator analyses 20](#_Toc123716274)

[Item 7. Results on sensitivity analyses 22](#_Toc123716275)

**Item 1. Full Pubmed search string**

(mental disorders[Mesh] OR mental illness[tiab] OR mental disorder[tiab] OR psychological disorder[tiab] OR psychological illness[tiab] OR psychological disease[tiab] OR psychiatric disorder[tiab] OR psychiatric illness[tiab] OR psychiatric disease[tiab] OR mood[tiab] OR anxiety[tiab] OR anxiety disorders[tiab] OR agoraphobia[tiab] OR separation anxiety[tiab] OR neurocirculatory Asthenia[tiab] OR neurotic Disorders[tiab] OR obsessive-compulsive[tiab] OR hoarding disorder[tiab] OR panic disorder[tiab] OR phobic disorders[tiab] OR social phobia[tiab] OR bipolar and related disorders[tiab] OR bipolar[tiab] OR disruptive disorders[tiab] OR impulse control[tiab] OR conduct disorders[tiab] OR firesetting behavior[tiab] OR gambling[tiab] OR trichotillomania[tiab] OR dissociative disorders[tiab] OR dissociative identity disorder[tiab] OR elimination disorders[tiab] OR encopresis[tiab] OR enuresis[tiab] OR diurnal enuresis[tiab] OR nocturnal enuresis[tiab] OR feeding and eating disorders[tiab] OR eating disorders[tiab] OR anorexia nervosa[tiab] OR binge-eating[tiab] OR bulimia nervosa[tiab] OR feeding and eating disorders of childhood[tiab] OR female athlete triad syndrome[tiab] OR food addiction[tiab] OR night eating[tiab] OR pica[tiab] OR mood disorders[tiab] OR cyclothymic disorder[tiab] OR depressive disorder[tiab] OR depression[tiab] OR postpartum depression[tiab] OR major depressive disorder[tiab] OR treatment-resistant depressive disorder[tiab] OR dysthymic disorder[tiab] OR premenstrual dysphoric disorder[tiab] OR seasonal affective disorder[tiab] OR motor disorders[tiab] OR neurocognitive disorders[tiab] OR amnesia[tiab] OR anterograde amnesia[tiab] OR retrograde amnesia[tiab] OR transient global amnesia[tiab] OR cognition disorders[tiab] OR auditory perceptual disorders[tiab] OR cognitive dysfunction OR Huntington disease[tiab] OR consciousness disorders[tiab] OR delirium[tiab] OR emergence delirium[tiab] OR dementia[tiab] OR AIDS dementia complex[tiab] OR Alzheimer[tiab] OR primary progressive aphasia[tiab] OR primary progressive nonfluent aphasia[tiab] OR Creutzfeldt-Jakob[tiab] OR vascular dementia[tiab] OR multi-infarct dementia[tiab] OR diffuse neurofibrillary tangles with calcification[tiab] OR frontotemporal lobar degeneration[tiab] OR frontotemporal dementia[tiab] OR Pick disease of the brain[tiab] OR Kluver-Bucy syndrome[tiab] OR Lewy Body disease[tiab] OR acquired dyslexia[tiab] OR pure alexia[tiab] OR neurodevelopmental disorders[tiab] OR attention deficit and disruptive behavior disorders[tiab] OR ADHD[tiab] OR ADD[tiab] OR attention deficit disorder with hyperactivity[tiab] OR conduct disorder[tiab] OR child behavior disorders[tiab] OR pervasive child development disorders[tiab] OR autism[tiab] OR Asperger syndrome[tiab] OR autistic[tiab] OR communication disorders[tiab] OR language disorders[tiab] OR agraphia[tiab] OR anomia[tiab] OR dyslexia[tiab] OR language development disorders[tiab] OR speech disorders[tiab] OR aphasia[tiab] OR Broca aphasia[tiab] OR conduction aphasia[tiab] OR primary progressive aphasia[tiab] OR primary progressive nonfluent aphasia[tiab] OR Wernicke aphasia[tiab] OR articulation disorders[tiab] OR dysarthria[tiab] OR echolalia[tiab] OR mutism[tiab] OR stuttering[tiab] OR learning disorders[tiab] OR dyscalculia[tiab] OR acquired dyslexia[tiab] OR developmental disabilities[tiab] OR intellectual disability[tiab] OR learning disorders[tiab] OR motor skills disorders[tiab] OR mutism[tiab] OR reactive attachment disorder[tiab] OR childhood schizophrenia[tiab] OR stereotypic movement disorder[tiab] OR tic disorders[tiab] OR Tourette syndrome[tiab] OR neurotic disorders[tiab] OR paraphilic disorders[tiab] OR exhibitionism[tiab] OR fetishism[tiab] OR masochism[tiab] OR pedophilia[tiab] OR sadism[tiab] OR transvestism[tiab] OR voyeurism[tiab] OR personality disorder[tiab] OR antisocial personality disorder[tiab] OR borderline personality disorder[tiab] OR compulsive personality disorder[tiab] OR dependent personality disorder[tiab] OR histrionic personality disorder[tiab] OR hysteria[tiab] OR paranoid personality disorder[tiab] OR passive-aggressive personality disorder[tiab] OR schizoid personality disorder[tiab] OR schizotypal personality disorder[tiab] OR schizophrenia spectrum and other psychotic disorders[tiab] OR schizophrenia spectrum[tiab] OR psychotic affective disorders[tiab] OR Capgras syndrome[tiab] OR delusional parasitosis[tiab] OR Morgellons disease[tiab] OR paranoid disorders[tiab] OR psychotic disorders[tiab] OR substance-induced psychoses[tiab] OR alcoholic psychoses[tiab] OR schizophrenia[tiab] OR catatonic schizophrenia[tiab] OR disorganized schizophrenia[tiab] OR paranoid schizophrenia[tiab] OR shared paranoid disorder[tiab] OR psychological sexual dysfunctions[tiab] OR dyspareunia[tiab] OR erectile dysfunction[tiab] OR gender dysphoria[tiab] OR premature ejaculation[tiab] OR sexual and gender disorders[tiab] OR vaginismus[tiab] OR sleep wake disorders[tiab] OR dyssomnias[tiab] OR sleep deprivation[tiab] OR  circadian rhythm sleep disorders[tiab] OR jet lag syndrome[tiab] OR intrinsic sleep disorders[tiab] OR disorders of excessive somnolence[tiab] OR idiopathic hypersomnolence[tiab] OR Kleine-Levin syndrome[tiab] OR narcolepsy[tiab] OR cataplexy[tiab] OR restless legs syndrome[tiab] OR sleep initiation and maintenance disorders[tiab] OR parasomnias[tiab] OR nocturnal myoclonus syndrome[tiab] OR nocturnal paroxysmal dystonia[tiab] OR REM sleep parasomnias[tiab] OR REM sleep behavior disorder[tiab] OR sleep paralysis[tiab] OR sleep arousal disorders[tiab] OR night terrors[tiab] OR somnambulism[tiab] OR sleep bruxism[tiab] OR sleep-wake transition disorders[tiab] OR somatoform disorders[tiab] OR body dysmorphic[tiab] OR conversion disorder[tiab] OR factitious disorders[tiab] OR Munchausen syndrome[tiab] OR Munchausen syndrome by proxy[tiab] OR hypochondriasis[tiab] OR neurasthenia[tiab] OR substance-related disorders[tiab] OR addiction[tiab] OR alcohol-related disorders[tiab] OR alcohol amnestic disorder[tiab] OR alcoholic Korsakoff syndrome[tiab] OR alcohol withdrawal delirium[tiab] OR alcoholic intoxication[tiab] OR alcoholism [tiab] OR binge drinking[tiab] OR alcoholic psychoses[tiab] OR Wernicke encephalopathy[tiab] OR amphetamine-related disorders[tiab] OR cocaine-related disorders[tiab] OR inhalant abuse[tiab] OR marijuana abuse[tiab] OR marijuana use[tiab] OR neonatal abstinence syndrome[tiab] OR opioid-related disorders[tiab] OR heroin dependence[tiab] OR morphine dependence[tiab] OR opium dependence[tiab] OR phencyclidine abuse[tiab] OR substance-induced psychoses[tiab] OR intravenous substance abuse[tiab] OR oral substance abuse[tiab] OR substance withdrawal syndrome[tiab] OR tobacco use disorder[tiab] OR trauma and stressor related disorders[tiab] OR adjustment disorders[tiab] OR traumatic stress disorders[tiab] OR battered child syndrome[tiab] OR combat disorders[tiab] OR psychological trauma[tiab] OR post-traumatic stress[tiab] OR acute traumatic stress[tiab] OR PTSD[tiab]) **AND** ("telemedicine"[Mesh] OR "telenursing"[Mesh] OR "user-computer interface"[Mesh] OR "multimedia"[Mesh] OR "cell phone"[Mesh] OR "public health informatics"[Mesh] OR "medical informatics"[Mesh] OR "nursing informatics"[Mesh] OR "computers, handheld"[Mesh] OR "mobile applications"[Mesh] OR "internet"[Mesh] OR "patient portals"[Mesh] OR econsult*[tiab] OR e-treat*[tiab] OR e-therap*[tiab] OR e-consult*[tiab] OR ediagnos*[tiab] OR e diagnos*[tiab] OR mobile health*[tiab] OR mhealth*[tiab] OR m health*[tiab] OR telehealth*[tiab] OR tele health[tiab] OR remote consult*[tiab] OR teleconsult*[tiab] OR tele consult*[tiab] OR telenursing[tiab] OR tele nursing[tiab] OR telediagnos*[tiab] OR tele diagnos*[tiab] OR telemedic*[tiab] OR tele medic*[tiab] OR telemonitor*[tiab] OR tele monitor*[tiab] OR ehealth*[tiab] OR e-health*[tiab] OR telecare[tiab] OR tele care[tiab] OR digital health[tiab] OR app[tiab] OR apps[tiab] OR smartphone*[tiab] OR phone application*[tiab] OR telephone application*[tiab] OR mobile application*[tiab] OR mobile technolog*[tiab] OR health technolog*[tiab] OR health application*[tiab] OR internet*[tiab] OR world wide web*[tiab] OR webportal*[tiab] OR web portal*[tiab] OR patient portal*[tiab] OR ipad[tiab] OR ipads[tiab] OR sms[tiab] OR mms[tiab] OR text messag*[tiab] OR ussd[tiab] OR pda[tiab] OR laptop*[tiab] OR palmtop*[tiab] OR palm top*[tiab] OR personal digital assistant*[tiab] OR telecounsel*[tiab] OR tele counsel*[tiab] OR remote counsel*[tiab] OR distance consult*[tiab] OR distance counsel*[tiab] OR distant consult*[tiab] OR patient monitoring[tiab] OR interactive voice response*[tiab] OR multimedia[tiab] OR Mhapps[tiab] OR iphone*[tiab] OR android[tiab] OR game*[tiab] OR gaming[tiab] OR gamification[tiab] OR whatsapp*[tiab] OR e-coach*[tiab] OR wearable*[tiab] OR social media[tiab] OR online*[tiab] OR computer*[tiab] OR electronic*[tiab] OR digital*[tiab] OR "online social network"[tiab] OR "online social networks"[tiab] OR facebook[tiab] OR exergam*[tiab] OR serious gam*[tiab] OR personal health record*[tiab] OR personal electronic health record*[tiab] OR health kiosk*[tiab] OR internet-based[tiab] OR internet based[tiab] OR web-based[tiab] OR web based[tiab] OR iCBT[tiab] OR oCBT[tiab] OR teleconferenc*[tiab] OR tele conferenc*[tiab] OR tele-conferenc*[tiab] OR telephone*[tiab] OR e-counsel*[tiab] OR short message service[tiab] OR SMS[tiab] OR cell-phone[tiab] OR cellphone[tiab] OR cellular phone*[tiab] OR blended*[tiab] OR email*[tiab] OR e-mail*[tiab] OR video-guid*[tiab] OR videoguid*[tiab] OR video-mediated[tiab] OR video-based[tiab] OR videobased[tiab] OR video-deliver*[tiab] OR video-treat*[tiab] OR video-therap*[tiab] OR videothera*[tiab] OR video-intervention*[tiab] OR video-counsel*[tiab] OR video-assist*[tiab] OR video-conferenc*[tiab] OR videoconferenc*[tiab] OR video-monit*[tiab] OR videomonit*[tiab] OR video-communicat*[tiab] OR videocommunicat*[tiab] OR video-remind*[tiab] OR video-administered*[tiab] OR video-aided[tiab] OR video-application*[tiab] OR video-consult*[tiab] OR videoconsult*[tiab] OR video-enabled[tiab] OR Twitter[tiab] OR Facebook[tiab] OR Instagram[tiab] OR forum[tiab] OR chat*[tiab] OR virtual reality*[tiab] OR virtual-reality*[tiab] OR avatar*[tiab] OR Conversational agent*[tiab] OR virtual coach[tiab] OR virtual agent*[tiab] OR embodied agent*[tiab] OR avatar*[tiab] OR relational agent*[tiab] OR interactive agent*[tiab] OR virtual character*[tiab] OR virtual human*[tiab] OR virtual assistant*[tiab] OR tele-psychiatry[tiab] OR telepsychiatry[tiab] OR tele-guid*[tiab] OR teleguid*[tiab] OR tele-based[tiab] OR tele-deliver*[tiab] OR teledeliver*[tiab] OR tele-treat*[tiab] OR teletreat*[tiab] OR tele-therap*[tiab] OR telethera*[tiab] OR tele-intervention*[tiab] OR tele-assist*[tiab] OR tele-communicat*[tiab] OR telecommunicat*[tiab]) **AND** (cost-benefit analysis[Mesh] OR "cost effectiveness analysis"[tiab] OR "cost effectiveness analyses"[tiab] OR "cost effectiveness"[tiab] OR "cost effective"[tiab] OR "economic evaluation"[tiab] OR "economic evaluations"[tiab] OR "cost benefit"[tiab] OR "cost-benefit analysis"[tiab] OR "cost-benefit analyses"[tiab] OR "cost-benefit data"[tiab] OR "cost utility"[tiab] OR "cost-utility analysis"[tiab] OR "cost-utility analyses"[tiab] OR marginal analyses[tiab] OR marginal analysis[tiab] OR cost minimization[tiab] OR cost-minimization[tiab] OR cost impact[tiab] OR cost-impact[tiab] OR budget impact[tiab] OR budget-impact[tiab])

**Item 2. Data extraction items**

| **Category** | **Extracted item** |
| --- | --- |
| General | - Author |
|  | - Year of publication |
|  | - Journal |
|  | - Country |
|  | - Randomized controlled trial (yes/no) |
| Participants | - Recruitment (com=community/open/mass media; clin=clinical recruitment; scr=systematic screening of a predefined population; other) |
|  | - Sample size (total) |
|  | - Sample size (per condition) |
|  | - % female |
|  | - Mean age (standard deviation) |
|  | - Targeted mental disorder |
|  | - Diagnose (1=formal diagnosis; 2=self-report; 3=other, please specify) |
|  | - Instrument for diagnosis |
|  | - Inclusion criteria |
|  | - Exclusion criteria |
| Interventions | - Intervention frequency and duration (per condition) |
|  | - Follow-up (i.e., time between baseline and last follow-up assessment) |
|  | - Assessment time points of quality of life/utility |
|  | - Assessment time points of costs/health care use |
|  | - Intervention description (per condition) |
|  | - Type of guidance (1=fully automated or no guidance; 2=asynchronous or guidance not at the same time such as e-mail/written feedback; 3=synchronous or guidance at the same time such as chat, telephone and face-to-face) |
|  | - Intensity of guidance (0=less than once a week; 1=once a week; 2=more than once a week; 3=self-guided; 4=other, please specify) |
| Questionnaires and methods | - Intention to treat analyses (yes/no) |
|  | - Primary outcome + measurement instrument |
|  | - Instrument used for quality of life/utility |
|  | - Instrument used for costs/health care use |
|  | - Source of (health care) unit costs |
|  | - Currency + year of indexing |
|  | - Discounting (0=none; 1=cost and effects at the same percentage, please specify %; 2=costs and effects at different percentages, please specify both percentages) |
|  | - Perspective used (1=health care; 2=societal; 3=other, please specify) |
|  | - If societal perspective was used, method for assessing productivity losses (1=friction cost; 2=human capital; 3=other, please specify) |
| Outcomes | - QALYs (per condition) |
|  | - SD/SE/variance/confidence interval of QALYs (per condition) |
|  | - Delta QALY (i.e., QALYs intervention – QALYs control) |
|  | - SD/SE/variance/confidence interval of delta QALY |
|  | - Health care costs (per condition) |
|  | - SD/SE/variance/confidence interval of health care costs (per condition) |
|  | - Delta health care costs (i.e., health care costs intervention – health care costs control) |
|  | - SD/SE/variance/confidence interval of delta health care costs |
|  | - Societal costs (per condition) |
|  | - SD/SE/variance/confidence interval of societal costs (per condition) |
|  | - Delta societal costs (i.e., societal costs intervention – societal costs control) |
|  | - SD/SE/variance/confidence interval of delta societal costs |
|  | - Incremental cost-effectiveness ratio |
|  | - Confidence interval of incremental cost-effectiveness ratio |
|  | - Cost-effectiveness plane for delta costs and delta QALYs provided (0=no; 1=yes) |
| Risk of bias | - Random sequence generation |
|  | - Allocation concealment |
|  | - Blinding of participants and personnel |
|  | - Blinding of outcome assessors |
|  | - Incomplete outcome data |
|  | - Selective reporting |
|  | - Other sources of bias |
| Quality of economic evaluation (CHEC) | - Is the study population clearly described? |
|  | - Are competing alternatives clearly described? |
|  | - Is a well-defined research question posed in answerable form? |
|  | - Is the economic study design appropriate to the stated objective? |
|  | - Is the chosen time horizon appropriate to include relevant costs and consequences? |
|  | - Is the actual perspective chosen appropriate? |
|  | - Are all important and relevant costs for each alternative identified? |
|  | - Are all costs measured appropriately in physical units? |
|  | - Are costs valued appropriately? |
|  | - Are all important and relevant outcomes for each alternative identified? |
|  | - Are all outcomes measured appropriately? |
|  | - Are outcomes valued appropriately? |
|  | - Is an incremental analysis of costs and outcomes of alternatives performed? |
|  | - Are all future costs and outcomes discounted appropriately? |
|  | - Are all important variables, whose values are uncertain, appropriately subjected to sensitivity analysis? |
|  | - Do the conclusions follow from the data reported? |
|  | - Does the study discuss the generalizability of the results to other settings and patient/client groups? |
|  | - Does the article indicate that there is no potential conflict of interest of study researcher(s) and funder(s)? |
|  | - Are ethical and distributional issues discussed appropriately? |

SD=standard deviation; SE=standard error; QALY=quality-of-life-adjusted life year

**Item 3. Formulas used for data preparation and final analyses.**

| **Outcome** | **Nr** | **Formula** |
| --- | --- | --- |
| **Data preparation** | | |
|  |  |  |
|  | 1 | ${Var}_{\Delta\mathrm{QALY}}={SD}_{\Delta\mathrm{QALY}}^{2}$ |
|  | 2 | ${Var}_{\Delta\mathrm{QALY}}={SE}_{\Delta\mathrm{QALY}}^{2}$ |
|  | 3 | ${SE}_{\Delta\mathrm{QALY}}=\frac{UL-{Mean}_{\Delta\mathrm{QALY}}}{1.96}$ |
|  | 4 | ${Var}_{\Delta\mathrm{QALY}}=\frac{{SD}_{intervention}^{2}}{N_{intervention}}+\frac{{SD}_{control}^{2}}{N_{control}}$ |
|  | 5 | ${Var}_{\Delta\mathrm{QALY}}={SE}_{intervention}^{2}+{SE}_{control}^{2}$ |
|  | 6 | ${Covariance}_{(\Delta QALY, \Delta Costs)}={SD}_{\Delta QALY}*{SD}_{\Delta Costs}*r_{(\Delta QALY, \Delta Costs)}$ |
|  | 7 | ${INB}_{study}=k*\Delta QALY-\Delta Costs$  Where k is society’s willingness to pay for one QALY |
| **Pooling studies** | | |
|  | 8 | ${INB}_{pooled}=\sum{(weight}_{study}*{INB}_{study})$ |
|  | 9 | ${weight}_{study}=\frac{1}{{Var(INB}_{study}) + \tau^{2}}$ |
|  | 10 | ${Var(INB}_{study})=\frac{1 / (k^{2}* {Var}_{\Delta\mathrm{QALY}}+{Var}_{\Delta\mathrm{Costs}}-2*k*covar(\Delta QALY, \Delta Costs))}{\sum(1 / {Var(INB}_{study})}$ |
|  | 11 | $\tau^{2}=\frac{Q-(s-1)}{\sum{(weight}_{study})*\frac{\sum{(weight}_{study})}{{\sum{(weight}_{study}}^{2})}}$  Where s is the number of included studies/comparisons, Q is the Cochran statistic and τ^2^=0 if Q < s – 1 |
| **Heterogeneity** | | |
|  | 12 | $Cochran Q=\sum_{s-1}^{s} \frac{1}{Var\left( {INB}_{study} \right)}*\left( {INB}_{study}-{INB}_{pooled} \right)^{2}$ |
|  |  | Where Q=0 if Q < s – 1 |
|  | 13 | $I^{2}=\frac{Q-\left( s-1 \right)}{Q}*100\%$ |

Note. The formula used to calculate delta QALY can also be used to calculate delta costs.

INB=incremental net benefit; Nr=reference number; QALY=quality adjusted life year; SD=standard deviation; SE=standard error; UL=upper limit; Var=variance

**Item 4. Example of the estimation of the covariance between delta QALY and delta costs with the use of Webplot Digitizer**


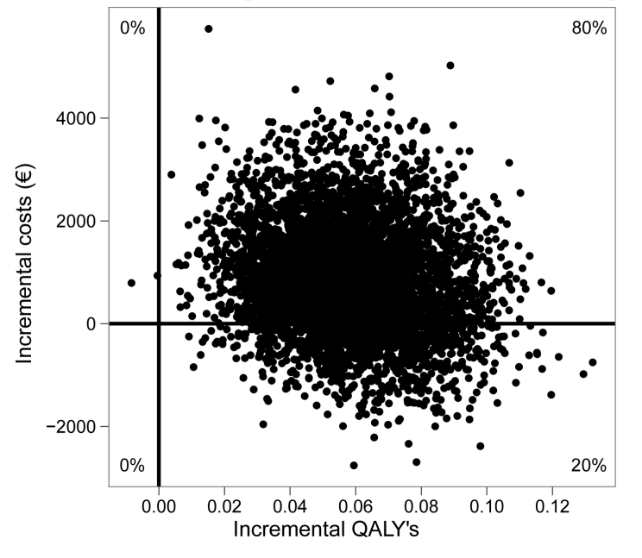
**[Step 1]**

Save the target cost-effectiveness plane with delta QALYs (x-axis) and delta costs (y-axis) as an image.

**[Step 2]**

Upload the image to Webplot Digitizer (<https://automeris.io/WebPlotDigitizer/>).

Calibrate x and y values, and select areas to reverse engineer individual data points.

**[Step 3]**

Run the application and download individual data points into a .csv format.

**[Step 4]**

Calculate the covariance and, if necessary, the standard deviations of delta QALY and delta costs using the appropriate Excel functions: “=COVARIANCE.S(range delta QALYs; range delta costs)” and “=STDEV.S(range)”.


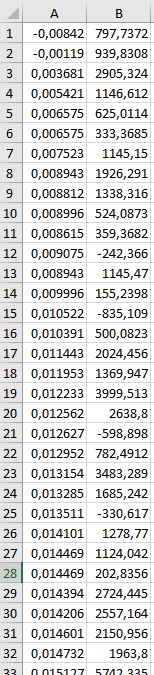

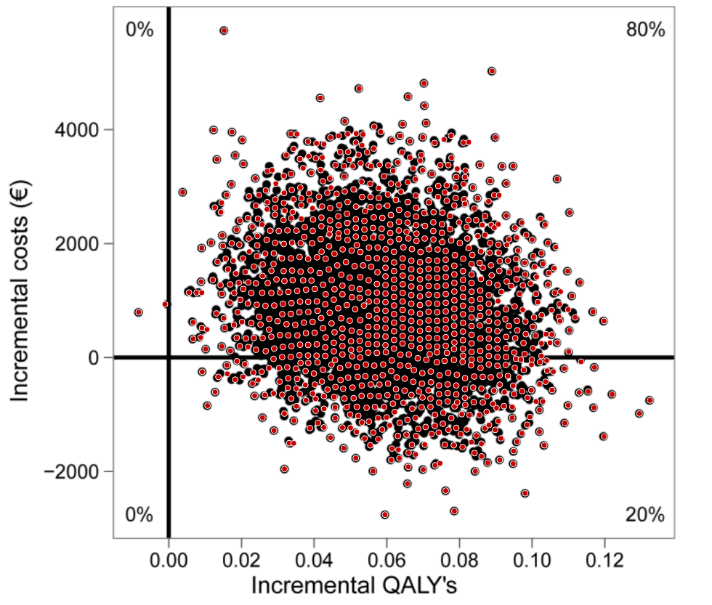


**Item 5. Excluded studies with reasons for exclusion**

| **#** | **Author (year of publication)** | **Title** | **Reason for exclusion** |
| --- | --- | --- | --- |
| 1 | Adewuya et al. (2019) | The effectiveness and acceptability of mobile telephone adherence support for management of depression in the Mental Health in Primary Care (MeHPriC) project, Lagos, Nigeria: A pilot cluster randomised controlled trial | Wrong outcomes |
| 2 | Andersson et al. (2011) | Cost-effectiveness of internet-based cognitive behavior therapy for irritable bowel syndrome: results from a randomized controlled trial | Wrong patient population |
| 3 | Andersson et al. (2015) | Cost-effectiveness of an internet-based booster program for patients with obsessive-compulsive disorder: Results from a randomized controlled trial | Wrong outcomes |
| 4 | Andersson et al. (2015) | Cost-effectiveness of internet-based cognitive behavior therapy for obsessive-compulsive disorder: Results from a randomized controlled trial | Wrong outcomes |
| 5 | Angus et al. (2019) | Cost-effectiveness of strategies to improve delivery of brief interventions for heavy drinking in primary care: results from the ODHIN trial | Wrong study design |
| 6 | Axelsson et al. (2018) | Cost-effectiveness and long-term follow-up of three forms of minimal-contact cognitive behaviour therapy for severe health anxiety: Results from a randomised controlled trial | Wrong outcomes |
| 7 | Bergstrom et al. (2010) | Internet-versus group-administered cognitive behaviour therapy for panic disorder in a psychiatric setting: a randomised trial | Wrong outcomes |
| 8 | Bischof et al. (2010) | Stepped-care intervention for alcohol problems: A cost-effective approach for brief interventions in primary care? | Study protocol |
| 9 | Blankers et al. (2012) | Clinical outcomes and economic evaluation of internet-based interventions for harmful alcohol use: a pragmatic randomized trial | Duplicate study |
| 10 | Blankers et al. (2012) | Economic evaluation of internet-based interventions for harmful alcohol use alongside a pragmatic randomized controlled trial | Wrong comparator |
| 11 | Boege et al. (2015) | Cost-effectiveness of intensive home treatment enhanced by inpatient treatment elements in child and adolescent psychiatry in Germany: A randomised trial | Wrong outcomes |
| 12 | Bogosian et al. (2021) | Acceptability and Feasibility of a Mindfulness Intervention Delivered via Videoconferencing for People With Parkinson's | Wrong outcomes |
| 13 | Bolier et al. (2014) | Cost-effectiveness of online positive psychology: Randomized controlled trial | Wrong outcomes |
| 14 | Botha et al. (2018) | Brief Report: A Randomized Control Trial Assessing the Influence of a Telephone-based Intervention on Readmissions for Patients with Severe Mental Illness in a Developing Country | Wrong outcomes |
| 15 | Brabyn et al. (2016) | The second Randomised Evaluation of the Effectiveness, cost-effectiveness and Acceptability of Computerised Therapy (REEACT-2) trial: does the provision of telephone support enhance the effectiveness of computer-delivered cognitive behaviour therapy? A randomised controlled trial | Wrong comparator |
| 16 | Budney et al. (2015) | Computer-assisted behavioral therapy and contingency management for cannabis use disorder | Wrong outcomes |
| 17 | Calhoun et al. (2016) | Comparative effectiveness of an Internet-based smoking cessation intervention versus clinic-based specialty care for veterans | Wrong outcomes |
| 18 | Celano et al. (2015) | Cost-effectiveness of a collaborative care depression and anxiety treatment program in patients with acute cardiac illness | Wrong intervention |
| 19 | Chalder et al. (2012) | A pragmatic randomised controlled trial to evaluate the cost-effectiveness of a physical activity intervention as a treatment for depression: the treating depression with physical activity (TREAD) trial | Wrong intervention |
| 20 | Chan et al. (2008) | Depression and comorbid PTSD in veterans: Evaluation of collaborative care programs and impact on utilization and costs | Wrong outcomes |
| 21 | ChoiYoo et al. (2014) | Cost effectiveness of telecare management for pain and depression in patients with cancer: results from a randomized trial | Wrong outcomes |
| 22 | Compen et al. (2017) | Face-to-face versus individual internetbased MBCT versus TAU for distressed cancer patients: The BeMind study | Conference abstract |
| 23 | Crow et al. (2009) | The cost effectiveness of cognitive behavioral therapy for bulimia nervosa delivered via telemedicine versus face-to-face | Wrong outcomes |
| 24 | Davidson et al. (2013) | Centralized, Stepped, Patient Preference-Based Treatment for Patients With Post-Acute Coronary Syndrome Depression CODIACS Vanguard Randomized Controlled Trial | Wrong outcomes |
| 25 | De Boer et al. (2014) | A randomized controlled trial of an Internet-based cognitive-behavioural intervention for non-specific chronic pain: An effectiveness and cost-effectiveness study | Wrong outcomes |
| 26 | De Bruin et al. (2016) | Cost-Effectiveness of Group and Internet Cognitive Behavioral Therapy for Insomnia in Adolescents: Results from a Randomized Controlled Trial | Wrong comparator |
| 27 | De Graaf et al. (2011) | One-year follow-up results of unsupported online computerized cognitive behavioural therapy for depression in primary care: A randomized trial | Wrong outcomes |
| 28 | Dear et al. (2020) | A Cost-effectiveness Analysis of an Internet-delivered Pain Management Program Delivered With Different Levels of Clinician Support: Results From a Randomised Controlled Trial | Wrong patient population |
| 29 | Delgadillo et al. (2017) | Improving the efficiency of psychological treatment using outcome feedback technology | Wrong study design |
| 30 | Dieng et al. (2013) | A randomised controlled trial of a psycho-educational intervention for melanoma survivors at high risk of developing new primary disease | Study protocol |
| 31 | Donohue et al. (2012) | 12-Month cost-effectiveness of telephonedelivered collaborative care for treating post-CABG depression | Conference abstract |
| 32 | Donohue et al. (2014) | Twelve-month cost-effectiveness of telephone-delivered collaborative care for treating depression following CABG surgery: a randomized controlled trial | Wrong intervention |
| 33 | Dorstyn et al. (2012) | Effectiveness of telephone counseling in managing psychological outcomes after spinal cord injury: a preliminary study | Wrong outcomes |
| 34 | Downe-Wamboldt et al. (2007) | The effects and expense of augmenting usual cancer clinic care with telephone problem-solving counseling | Wrong outcomes |
| 35 | Drost et al. (2016) | A Web-Based Computer-Tailored Alcohol Prevention Program for Adolescents: Cost-Effectiveness and Intersectoral Costs and Benefits | Wrong outcomes |
| 36 | Duarte et al. (2014) | Computerised Cognitive Behaviour Therapy for Depression Management: A Cost-Effectiveness Analysis | Duplicate study |
| 37 | Duarte et al. (2014) | Computerised cognitive behaviour therapy for depression management: A cost-effectiveness analysis | Conference abstract |
| 38 | Dunlap et al. (2019) | Screening and Intervention for Suicide Prevention: A Cost-Effectiveness Analysis of the ED-SAFE Interventions | Wrong outcomes |
| 39 | Ebert et al. (2018) | A health economic outcome evaluation of an internet-based mobile-supported stress management intervention for employees | Wrong patient population |
| 40 | Egede et al. (2018) | Cost-Effectiveness of Behavioral Activation for Depression in Older Adult Veterans: In-Person Care Versus Telehealth | Wrong outcomes |
| 41 | El Alaoui et al. (2017) | Does internet-based cognitive behaviour therapy reduce healthcare costs and resource use in treatment of social anxiety disorder? A cost-minimisation analysis conducted alongside a randomised controlled trial | Wrong outcomes |
| 42 | Esmaeili et al. (2020) | Budget Impact Analysis of a Computer-Delivered Brief Alcohol Intervention in Veterans Affairs (VA) Liver Clinics: A Randomized Controlled Trial | Wrong outcomes |
| 43 | Everitt et al. (2019) | Therapist telephone-delivered CBT and web-based CBT compared with treatment as usual in refractory irritable bowel syndrome: the ACTIB three-arm RCT | Wrong patient population |
| 44 | Fabian et al. (2017) | Cost-effectiveness of Therapist-guided Internet-delivered Cognitive Behavior Therapy for Pediatric Obsessive-Compulsive Disorder | Duplicate study |
| 45 | Fortney et al. (2011) | A budget impact analysis of telemedicine-based collaborative care for depression | Wrong outcomes |
| 46 | Garrido et al. (2017) | Computer-assisted cognitive remediation therapy in schizophrenia: Durability of the effects and cost-utility analysis | Wrong outcomes |
| 47 | Gerhards et al. (2011) | Economic evaluation of online computerized cognitive behavioural therapy without support for depression in primary care: A randomized trial | Conference abstract |
| 48 | Gidding et al. (2018) | PsyScan e-tool to support diagnosis and management of psychological problems in general practice: a randomised controlled trial | Wrong outcomes |
| 49 | Godfrey et al. (2005) | Cost effectiveness of treatment for alcohol problems: Findings of the randomised UK alcohol treatment trial (UKATT) | Wrong intervention |
| 50 | Grafe et al. (2017) | Internet based treatment of depressive symptoms-a health economic evaluation of costs and benefits | Conference abstract |
| 51 | Gräfe et al. (2019) | Health economic evaluation of a web-based intervention for depression: the EVIDENT-trial, a randomized controlled study | Wrong outcomes |
| 52 | Gräfe et al. (2020) | Health economic evaluation of an internet intervention for depression (deprexis), a randomized controlled trial | Wrong outcomes |
| 53 | Gryczynski et al. (2021) | Computer- vs. nurse practitioner-delivered brief intervention for adolescent marijuana, alcohol, and sex risk behaviors in school-based health centers | Wrong outcomes |
| 54 | Hange et al. (2017) | The impact of internet-based cognitive behavior therapy on work ability in patients with depression - a randomized controlled study | Wrong outcomes |
| 55 | Havard et al. (2012) | Randomized Controlled Trial of Mailed Personalized Feedback for Problem Drinkers in the Emergency Department: the Short-Term Impact | Wrong intervention |
| 56 | Hedman et al. (2011) | Cost-effectiveness of Internet-based cognitive behavior therapy vs. cognitive behavioral group therapy for social anxiety disorder: results from a randomized controlled trial | Wrong outcomes |
| 57 | Hedman et al. (2013) | Cost-effectiveness and long-term effectiveness of internet-based cognitive behaviour therapy for severe health anxiety | Wrong outcomes |
| 58 | Hedman et al. (2014) | Clinical effectiveness and cost-effectiveness of Internet- vs. group-based cognitive behavior therapy for social anxiety disorder: 4-year follow-up of a randomized trial | Wrong outcomes |
| 59 | Hedman et al. (2014) | Clinical effectiveness and cost-effectiveness of Internet- vs. group-based cognitive behavior therapy for social anxiety disorder: 4-year follow-up of a randomized trial | Duplicate study |
| 60 | Hedman et al. (2016) | Cost effectiveness of internet-based cognitive behaviour therapy and behavioural stress management for severe health anxiety | Wrong outcomes |
| 61 | Hedman-Lagerlof et al. (2019) | Cost-Effectiveness and Cost-Utility of Internet-Delivered Exposure Therapy for Fibromyalgia: Results From a Randomized, Controlled Trial | Wrong patient population |
| 62 | Henderson et al. (2013) | Cost effectiveness of telehealth for patients with long term conditions (Whole Systems Demonstrator telehealth questionnaire study): nested economic evaluation in a pragmatic, cluster randomised controlled trial | Wrong patient population |
| 63 | Hollinghurst et al. (2009) | Effectiveness and Cost-Effectiveness of an Internet Based Cognitive Behavioural Psychotherapy for Depression: A Randomised Controlled Trial | Conference abstract |
| 64 | Hudson et al. (2017) | Tailored online cognitive behavioural therapy with or without therapist support calls to target psychological distress in adults receiving haemodialysis: A feasibility randomised controlled trial | Wrong study design |
| 65 | Isetta et al. (2015) | A bayesian cost-effectiveness analysis of a telemedicine-based strategy for the management of sleep apnea: A multicenter randomized controlled trial | Wrong outcomes |
| 66 | Jahoda et al. (2017) | Comparison of behavioural activation with guided self-help for treatment of depression in adults with intellectual disabilities: a randomised controlled trial | Wrong intervention |
| 67 | Jahoda et al. (2018) | Behavioural activation versus guided self-help for depression in adults with learning disabilities: the BeatIt RCT | Wrong intervention |
| 68 | Kafali et al. (2014) | Cost-effectiveness of a randomized trial to treat depression among Latinos | Wrong outcomes |
| 69 | Kaldo et al. (2008) | Internet versus group cognitive-behavioral treatment of distress associated with tinnitus: a randomized controlled trial | Wrong outcomes |
| 70 | Kamat et al. (2019) | Effect of video-assisted patient education on compliance with therapy, quality of life, psychomorbidity, and cost of illness in irritable bowel syndrome | Wrong patient population |
| 71 | Kemmeren et al. (2016) | The cost-effectiveness of blended cognitive therapy for depression, the e-compared study in the Netherlands | Study protocol |
| 72 | Kiluk et al. (2016) | Randomized Trial of Computerized Cognitive Behavioral Therapy for Alcohol Use Disorders: Efficacy as a Virtual Stand-Alone and Treatment Add-On Compared with Standard Outpatient Treatment | Wrong outcomes |
| 73 | Klein et al. (2018) | Economic Evaluation of an Internet-Based Preventive Cognitive Therapy With Minimal Therapist Support for Recurrent Depression: Randomized Controlled Trial | Wrong patient population |
| 74 | König et al. (2018) | Economic evaluation of cognitive behavioral therapy and Internet-based guided self-help for binge-eating disorder | Wrong comparator |
| 75 | Kooistra et al. (2019) | Cost and Effectiveness of Blended Versus Standard Cognitive Behavioral Therapy for Outpatients With Depression in Routine Specialized Mental Health Care: Pilot Randomized Controlled Trial | Wrong comparator |
| 76 | Kruger et al. (2014) | The cost-effectiveness of a theory-based online health behaviour intervention for new university students: an economic evaluation | Wrong patient population |
| 77 | Lalouni et al. (2018) | Clinical and Cost Effectiveness of Online Cognitive Behavioral Therapy in Children With Functional Abdominal Pain Disorders | Wrong patient population |
| 78 | Lavelle et al. (2018) | Cost-effectiveness of collaborative care for depression and PTSD in military personnel | Wrong intervention |
| 79 | Le et al. (2019) | The Cost-Effectiveness of an Internet Intervention to Facilitate Mental Health Help-Seeking by Young Adults: Randomized Controlled Trial | Wrong patient population |
| 80 | Lenhard et al. (2016) | Cost-effectiveness of internetdelivered cognitive-behavior therapy for obsessive-compulsive disorder: Results from a randomized controlled trial | Conference abstract |
| 81 | Lenhard et al. (2016) | Cost-effectiveness of internet-delivered cognitive behavior therapy for adolescent obsessive-compulsive disorder | Conference abstract |
| 82 | Lenhard et al. (2020) | Long-term outcomes of therapist-guided Internet-delivered cognitive behavior therapy for pediatric obsessive-compulsive disorder | Wrong outcomes |
| 83 | Littlewood et al. (2015) | A randomised controlled trial of computerised cognitive behaviour therapy for the treatment of depression in primary care: the Randomised Evaluation of the Effectiveness and Acceptability of Computerised Therapy (REEACT) trial | Duplicate study |
| 84 | Liu et al. (2003) | Cost-effectiveness of collaborative care for depression in a primary care veteran population | Wrong outcomes |
| 85 | Ljotsson et al. (2011) | Acceptability, effectiveness, and cost-effectiveness of internet-based exposure treatment for irritable bowel syndrome in a clinical sample: a randomized controlled trial | Wrong patient population |
| 86 | Lobban et al. (2020) | A web-based, peer-supported self-management intervention to reduce distress in relatives of people with psychosis or bipolar disorder: the REACT RCT | Wrong patient population |
| 87 | Lobban et al. (2020) | Clinical effectiveness of a web-based peer-supported self-management intervention for relatives of people with psychosis or bipolar (REACT): online, observer-blind, randomised controlled superiority trial | Wrong outcomes |
| 88 | Lokman et al. (2015) | Return-to-work intervention versus care as usual for sick listed employees with common mental disorders: Trial-based economic evaluation shows promise | Conference abstract |
| 89 | Lokman et al. (2017) | Complaint-Directed Mini-Interventions for Depressive Complaints: A Randomized Controlled Trial of Unguided Web-Based Self-Help Interventions | Wrong outcomes |
| 90 | Mayoral et al. (2017) | Economic Evaluation of a Guided and Unguided Internet-Based CBT Intervention for Major Depression: Results from a Multicentre Three-Armed Randomized Controlled Trial Conducted in Primary Care | Duplicate study |
| 91 | McCollister et al. (2016) | Cost-effectiveness analysis of a continuing care intervention for cocaine-dependent adults | Wrong outcomes |
| 92 | McCrone et al. (2004) | Cost-effectiveness of computerised cognitive-behavioural therapy for anxiety and depression in primary care: randomised controlled trial | Wrong outcomes |
| 93 | McCrone et al. (2007) | Cost-effectiveness of computer-aided behaviour therapy for obsessive-compulsive disorder | Wrong outcomes |
| 94 | Moayeri et al. (2018) | Cost-utility analysis of telephone-based cognitive behavior therapy in chronic obstructive pulmonary disease (COPD) patients with anxiety and depression comorbidities: an application for willingness to accept concept | Duplicate study |
| 95 | Moessner et al. (2014) | Cost-effectiveness of an internet-based aftercare intervention after inpatient treatment in a psychosomatic hospital | Wrong outcomes |
| 96 | Mohr et al. (2019) | A randomized noninferiority trial evaluating remotely-delivered stepped care for depression using internet cognitive behavioral therapy (CBT) and telephone CBT | Wrong comparator |
| 97 | Moradi-Lakeh et al. (2017) | Cost-effectiveness of aftercare services for people with severe mental disorders: an analysis parallel to a randomised controlled clinical trial in Iran | Wrong intervention |
| 98 | Moss-Morris et al. (2015) | A pilot randomized controlled trial of the clinical and cost effectiveness of a skype delivered group mindfulness intervention for distressed people with progressive multiple sclerosis | Conference abstract |
| 99 | Mouthaan et al. (2011) | Quality of Life and Cost-Effectiveness of a Brief Web-Based Early Intervention to Prevent PTSD in Traumatic Injury Patients | Study protocol |
| 100 | Noben et al. (2014) | Comparative cost-effectiveness of two interventions to promote work functioning by targeting mental health complaints among nurses: pragmatic cluster randomised trial | Wrong outcomes |
| 101 | Noben et al. (2015) | Comparative cost-effectiveness of two interventions to promote work functioning by targeting mental health complaints among nurses: Pragmatic cluster randomised trial | Conference abstract |
| 102 | Nordgren et al. (2014) | Effectiveness and cost-effectiveness of individually tailored Internet-delivered cognitive behavior therapy for anxiety disorders in a primary care population: a randomized controlled trial | Wrong outcomes |
| 103 | O’Connell et al. (2017) | Discrete event simulation modelling of long term cost-effectiveness of internet-based blended cognitive behavioural therapy for major depressive disorder: Extrapolati on of the e-compared randomised controlled trial | Conference abstract |
| 104 | Olmstead et al. (2010) | Cost-effectiveness of computer-assisted training in cognitive-behavioral therapy as an adjunct to standard care for addiction | Wrong outcomes |
| 105 | Olmstead et al. (2019) | Cost-effectiveness of Electronic- and Clinician-Delivered Screening, Brief Intervention, and Referral to Treatment for Women in Reproductive Health Centers | Wrong outcomes |
| 106 | Osborne et al. (2019) | Cost-effectiveness of internet-based cognitive-behavioural therapy for obsessive-compulsive disorder | Wrong study design |
| 107 | Painter et al. (2015) | Cost-Effectiveness of Collaborative Care for Depression in HIV Clinics | Wrong intervention |
| 108 | Painter et al. (2017) | Cost-Effectiveness of Telemedicine-Based Collaborative Care for Posttraumatic Stress Disorder | Wrong intervention |
| 109 | Pot-Kolder et al. (2020) | Cost-Effectiveness of Virtual Reality Cognitive Behavioral Therapy for Psychosis: Health-Economic Evaluation Within a Randomized Controlled Trial | Wrong intervention |
| 110 | Pyne et al. (2010) | Cost-effectiveness analysis of a rural telemedicine collaborative care intervention for depression | Wrong intervention |
| 111 | Pyne et al. (2015) | Cost-effectiveness of on-site versus off-site collaborative care for depression in rural FQHCs | Wrong intervention |
| 112 | Richards et al. (2016) | Clinical effectiveness and cost-effectiveness of collaborative care for depression in UK primary care (CADET): a cluster randomised controlled trial | Wrong intervention |
| 113 | Rollman et al. (2012) | The 12-month cost-effectiveness of telephone delivered collaborative care for post-CABG depression | Conference abstract |
| 114 | Ruskin et al. (2004) | Treatment outcomes in depression: comparison of remote treatment through telepsychiatry to in-person treatment | Wrong outcomes |
| 115 | Salisbury et al. (2017) | An evidence-based approach to the use of telehealth in long-term health conditions: development of an intervention and evaluation through pragmatic randomised controlled trials in patients with depression or raised cardiovascular risk | Wrong intervention |
| 116 | Schotanus-Dijkstra et al. (2018) | Towards sustainable mental health promotion: trial-based health-economic evaluation of a positive psychology intervention versus usual care | Wrong outcomes |
| 117 | Schubert et al. (2015) | Cost-effectiveness Analysis of a Telephone-based Managed Care Program for Mental Disorders from the Perspective of a Statutory Health Insurance. [German] | No English |
| 118 | Sembi et al. (2015) | Mums 4 Mums: Pilot randomised controlled trial of the clinical and cost-effectiveness of telephone peer support for postnatal depression | Conference abstract |
| 119 | Shepard et al. (2016) | Telephone-based continuing care counseling in substance abuse treatment: Economic analysis of a randomized trial | Wrong outcomes |
| 120 | Simon et al. (2001) | Cost-effectiveness of systematic depression treatment for high utilizers of general medical care | Wrong intervention |
| 121 | Simon et al. (2002) | Cost-effectiveness of a program to prevent depression relapse in primary care | Wrong outcomes |
| 122 | Simon et al. (2006) | Long-term effectiveness and cost of a systematic care program for bipolar disorder | Wrong outcomes |
| 123 | Simon et al. (2009) | Incremental benefit and cost of telephone care management and telephone psychotherapy for depression in primary care | Wrong outcomes |
| 124 | Smit et al. (2006) | Cost-effectiveness of preventing depression in primary care patients - Randomised trial | Wrong outcomes |
| 125 | Smit et al. (2013) | Cost-effectiveness and cost-utility of Internet-based computer tailoring for smoking cessation | Wrong patient population |
| 126 | Solomon et al. (2015) | e-CBT (myCompass), Antidepressant Medication, and Face-to-Face Psychological Treatment for Depression in Australia: A Cost-Effectiveness Comparison | Wrong study design |
| 127 | Spindler et al. (2010) | Telehealth in the parkinson's disease subspecialty clinic: The key to the patient-centered medical home | Conference abstract |
| 128 | Thase et al. (2020) | Improving Cost-effectiveness and Access to Cognitive Behavior Therapy for Depression: Providing Remote-Ready, Computer-Assisted Psychotherapy in Times of Crisis and Beyond | Wrong comparator |
| 129 | Thiart et al. (2016) | Internet-Based Cognitive Behavioral Therapy for Insomnia: A Health Economic Evaluation | Wrong outcomes |
| 130 | Titov et al. (2009) | Shyness programme: longer term benefits, cost-effectiveness, and acceptability | Wrong outcomes |
| 131 | Valimaki et al. (2017) | Short text messages to encourage adherence to medication and follow-up for people with psychosis (mobile.net): Randomized controlled trial in Finland | Wrong outcomes |
| 132 | Van Eeden et al. (2015) | An economic evaluation of an augmented cognitive behavioural intervention vs. computerized cognitive training for post-stroke depressive symptoms | Wrong intervention |
| 133 | Van Nispen et al. (2016) | Cost-effectiveness of stepped-care implemented in low vision rehabilitation to reduce depression and anxiety in vision impaired older adults | Wrong intervention |
| 134 | Van Spijker et al. (2012) | Reducing suicidal ideation: cost-effectiveness analysis of a randomized controlled trial of unguided web-based self-help | Wrong outcomes |
| 135 | Van Spijker et al. (2016) | Online self-help for persons with suicidal intentions: budget impact analysis | Wrong study design |
| 136 | Verdonck-De Leeuw et al. (2013) | Cost-evaluation of online guided self-help targeting psychological distress in cancer survivors | Conference abstract |
| 137 | Verdonck-De Leeuw et al. (2013) | Efficacy and cost-evaluation of web-based guided self-help targeting psychological distress in cancer survivors | Conference abstract |
| 138 | Watson et al. (2018) | Cost-Effectiveness of Internet-Based Cognitive-Behavioral Treatment for Bulimia Nervosa: Results of a Randomized Controlled Trial | Wrong comparator |
| 139 | Wijnen et al. (2018) | Complaint-Directed Mini-Interventions for Depressive Symptoms: A Health Economic Evaluation of Unguided Web-Based Self-Help Interventions Based on a Randomized Controlled Trial | Wrong outcomes |
| 140 | Wright et al. (2017) | Computerised cognitive-behavioural therapy for depression in adolescents: Feasibility results and 4-month outcomes of a UK randomised controlled trial | Duplicate study |
| 141 | Zhou et al. (2019) | Efficacy and cost-effectiveness of internet-based cognitive behavioral therapy for obsessive-compulsive disorder. [Chinese] | No English |

Note. Wrong outcomes can mean that no QALYs and/or costs were reported, that QALYs were reported but calculated inadequately or that included costs were not sufficient/appropriate.

**Item 6. Results on moderator analyses**

| **#** | **Subgroups** | ***N*** | **Cochrane *Q*** | ***I^2^* (95% CI)** | **Pooled INB** | **95% CI Pooled INB** | ***P*-value** |
| --- | --- | --- | --- | --- | --- | --- | --- |
| 1 | Health care perspective | 15 | *Q*(14)=14.2, *P*=.43 | 15.1% (0.0%; 64.0%) | $280 | $109; $451 | .001 |
|  | Societal perspective | 22 | *Q*(21)=22.5, *P*=.37 | 0.0% (0.0%; 0.01%) | $161 | $-247; $569 | .44 |
|  |  |  |  |  |  |  |  |
| 2 | Shorter than 12-month follow-up | 14 | *Q*(13)=5.6, *P*=.96 | 0.0% (0.0%; 0.03%) | $112 | $-194; $418 | .47 |
|  | 12-month follow-up or longer | 23 | *Q*(22)=30.3, *P*=.11 | 12.3% (0.0%; 58.3%) | $270 | $-14; $554 | .063 |
|  |  |  |  |  |  |  |  |
| 3 | Depression | 16 | *Q*(15)=13.6, *P*=.55 | 0.0% (0.0%; 0.03%) | $387 | $156; $618 | .001 |
|  | Anxiety | 7 | *Q*(6)=1.2, *P*=.98 | 0.0% (0.0%; 0.0%) | $644 | $227; $1061.82 | .002 |
|  | Alcohol or substance abuse | 5 | *Q*(4)=3.6, *P*=.46 | 11.6% (0.0%; 56.6%) | $-129 | $-448; $191 | .43 |
|  | Depression and anxiety simultaneously | 5 | *Q*(4)=6.7, *P*=.15 | 13.2% (0.0%; 60.3%) | $580 | $-584; $1744 | .33 |
|  | obsessive compulsive disorder | 2 | *Q*(1)=0.2, *P*=.68 | 0.0% (0.0%; 0.03%) | $253 | $-544; $1051 | .53 |
|  |  |  |  |  |  |  |  |
| 4 | Self-guided intervention | 10 | *Q*(9)=23.7, *P*=.005 | 45.2% (36.7%; 89.2%) | $169 | $-266; $604 | .45 |
|  | Guided intervention | 27 | *Q*(26)=13.1, *P=*.98 | 0.0% (0.0%; 0.0%) | $317 | $84; $550 | .008 |
|  |  |  |  |  |  |  |  |
| 5 | Self-guided intervention | 10 | *Q*(9)=23.7, *P=*.005 | 45.2% (36.7%; 89.2%) | $169 | $-266; $604 | .45 |
|  | Less than weekly guidance | 3 | *Q*(2)=0.7, *P*=.71 | 0.0% (0.0%; 0.02%) | $108 | $-618; $835 | .77 |
|  | Weekly guidance | 21 | *Q*(20)=9.9, *P*=.97 | 0.0% (0.0%; 0.02%) | $413 | $146; $680 | .002 |
|  | More than weekly guidance | 3 | *Q*(2)=0.2, *P*=.90 | 0.0% (0.0%; 0.02%) | $-67 | $-699; $565 | .84 |
|  |  |  |  |  |  |  |  |
| 6 | Asynchronous guidance | 11 | *Q*(10)=2.4, *P*=.99 | 0.0% (0.0%; 0.0%) | $375 | $-229; $979 | .22 |
|  | Synchronous guidance | 11 | *Q*(10)=8.0, *P*=.62 | 0.0% (0.0%; 0.02%) | $94 | $-335; $524 | .67 |
|  | Combination | 5 | *Q*(4)=1.1, *P*=.89 | 0.0% (0.0%; 0.03%) | $418 | $106; $730 | .009 |
|  |  |  |  |  |  |  |  |
| 7 | Open/mass media recruitment | 13 | *Q*(12)=6.2, *P*=.91 | 6.9% (0.0%; 16.7%) | $397 | $173; $621 | .001 |
|  | Recruitment by clinical referral | 20 | *Q*(19)=23.7, *P*=.21 | 1.7% (0.0%; 14.8%) | $138 | $-170; $446 | .38 |
|  | Other* | 4 | *Q*(3)=4.4, *P*=.22 | 0.0% (0.0%; 0.01%) | $91 | $-1241; $1423 | .89 |
|  |  |  |  |  |  |  |  |
| 8 | Formal diagnosis for inclusion | 13 | *Q*(12)=11.5, *P*=.48 | 0.0% (0.0%; 0.01%) | $311 | $-192; $814 | .23 |
|  | Self-reported symptoms for inclusion | 24 | *Q*(23)=25.5, *P*=.32 | 12.4% (0.0%; 58.6%) | $235 | $38; $432 | .02 |
|  |  |  |  |  |  |  |  |
| 9 | 4-8 weeks intervention duration | 17 | *Q*(16)=10.3, *P*=.85 | 4.3% (0.0%; 31.1%) | $410 | $168; $652 | <.001 |
|  | 9-12 weeks intervention duration | 13 | *Q*(12)=13.5, *P*=.33 | 0.0% (0.0%; 0.03%) | $250 | $-36; $535 | .09 |
|  | Duration more than 12 weeks | 3 | *Q*(2)=0.9, *P*=.65 | 0.0% (0.0%; 0.01%) | $-107 | $-979; $765 | .81 |
|  | Undefined intervention duration | 4 | *Q*(3)=6.6, *P*=.09 | 25.6% (0.0%; 77.4%) | $-244 | $-960; $471 | .50 |
|  |  |  |  |  |  |  |  |
| 10 | Care-as-usual control condition | 32 | *Q*(31)=33.7, *P*=.34 | 7.9% (0.0%; 46.1%) | $261 | $77; $445 | .005 |
|  | Attention control condition | 5 | *Q*(4)=3.0, *P*=.55 | 0.0% (0.0%; 0.01%) | $64 | $-617; $745 | .85 |

CI=confidence interval; INB=incremental net benefit.

*Studies falling under this category used a either both open recruitment and clinical referral or screened a specific population.

**Item 7. Results on sensitivity analyses**

| **#** | **Subgroups** | ***N*** | **Cochrane *Q*** | ***I^2^* (95% CI)** | **Pooled INB** | **95% CI Pooled INB** | ***P*-value** |
| --- | --- | --- | --- | --- | --- | --- | --- |
| 1 | High CHEC list quality rating | 24 | *Q*(23)=18.6, *P*=.72 | 0.8% (0.0%; 7.6%) | $253 | $43; $463 | .018 |
|  | Low CHEC list quality rating | 13 | *Q*(12)=18.5, *P*=.10 | 26.0% (0.0%; 77.8%) | $197 | $-155; $548 | .27 |
|  |  |  |  |  |  |  |  |
| 2 | Low risk of bias rating | 8 | *Q*(7)=3.4, *P*=.84 | 0.0% (0.0%; 0.01%) | $244 | $-555; $1042 | .55 |
|  | Medium risk of bias rating | 8 | *Q*(7)=13.0, *P*=.072 | 0.0% (0.0%; 0.01%) | $134 | $-237; $505 | .48 |
|  | High risk of bias rating | 21 | *Q*(20)=20.1, *P*=.45 | 9.3% (0.0%; 50.8%) | $287 | $113; $461 | .001 |
|  |  |  |  |  |  |  |  |
| 3 | QALY valued at $20,000 | 37 | *Q*(36)=36.1, *P*=.46 | 5.3% (0.0%; 35.6%) | $145 | $56 $234 | .001 |
|  |  |  |  |  |  |  |  |
| 4 | QALY valued at $80,000 | 37 | *Q*(36)=40.0, *P*=.30 | 12.9% (0.0%; 59.6%) | $431 | $115; $747 | .008 |
|  |  |  |  |  |  |  |  |
| 5 | Studies with directly calculated covariances | 12 | *Q*(11)=7.0, *P*=.80 | 0.0% (0.0%; 0.02%) | $264 | $-167; $694 | .23 |
|  | Studies with indirectly calculated covariances | 25 | *Q*(24)=30.1, *P*=.18 | 14.0% (0.0%; 61.8%) | $236 | $27; $445 | .03 |

CI=confidence interval; INB=incremental net benefit.
